# A distribution-based selective optimization method for eliminating periodic defects in harmonic signals

**DOI:** 10.1016/j.ymssp.2022.109781

**Published:** 2023-02

**Authors:** Qing-Yuan Xin, Yong-Chen Pei, Huiqi Lu, David Clifton, Bin Wang, Chuan Qu, Lu-Lu Wang, Meng-Yan Luo

**Affiliations:** a School of Mechanical and Aerospace Engineering, Jilin University, Nanling Campus, Changchun 130025, China; b Institute of Biomedical Engineering, Department of Engineering Science, University of Oxford, Oxford OX3 7DQ, United Kingdom; c Oxford-Suzhou Centre for Advanced Research, Suzhou 215000, China

**Keywords:** Harmonic signal, Periodic defect, Defect elimination, Selective optimization fitting, Error distribution statistics

## Abstract

Due to environmental interference and defects in measured objects, measurement signals are frequently affected by unpredictable noise and periodic defects. Moreover, there is a lack of effective methods for accurately distinguishing defect components from measurement signals. In this study, a distribution-based selective optimisation method (SOM) is proposed to mitigate the effects of noise and defect components. The SOM can be seen as a binary- or multiple-class signal classifier based on an error distribution, which can simultaneously eliminate periodic defect components of measurement signals and proceed with signal-fitting regression. The effectiveness, accuracy, and feasibility of the SOM are verified in theoretical and realworld measurement settings. Based on theoretical simulations under various parameter conditions, some criteria for selecting operation variables among a selection of parameter conditions are explained in detail. The proposed method is capable of separating defect components from measurement signals while also achieving a satisfactory fitting curve for the measurement signals. The proposed SOM has broad application prospects in signal processing and defect detection for mechanical measurements, electronic filtering, instrumentation, part maintenance, and other fields.

## 1 Introduction

Measurement data are often collected through scientific and engineering experiments. The relationships between measurement data *y* and independent variables *x* are examined to analyse signal characteristics, which can be denoted as an approximate expression of discrete points (*x_i_*, *y_i_*) or fitting function *f*(*x*_i_) in signal processing. Conventional fitting methods for measurement signals include the original least squares (OLS) [1], fast Fourier transform (FFT) [2], principal component analysis (PCA) [3], wavelet transform (WT) [4,5], least mean squares (LMS) [6,7], and maximum likelihood estimation (MLE) [8]. However, few methods can efficiently handle environmental interference in measurement signals while fitting discrete points (*x_i_*, *y_i_*). Such environmental interference include noise and mechanical defects. To provide an accurate function *f*(*x*_i_) for data interfered with random and non-random interference, scientists worldwide have optimised conventional fitting methods and have provided a few approaches to improve fitting accuracy and computational efficiency.

For a measurement signal with a smooth waveform, many scientists research signal characteristic based on improved fitting methods. Brown et al. [9] proposed a signal-period recognition method based on a feedback error system. Tenneti et al. [10] proposed a Nested Periodic Matrix method for detecting signal periods using nested periodic matrices. Qiu et al. [11] presented an optimisation approach to obtain the exact frequency characteristics of harmonic signals. Tan et al. [12] obtained the signal frequency using a linear model of the frequency measurement based on least-squares regression analysis. Gurubelli et al. [13] developed a method for estimating the signal frequency of sampled sinusoidal signals. These studies have helped improve the fitting accuracy of the measurement signals. However, these methods can neither deal with noise with large amplitudes nor handle multiple defects.

For measurement signals with noise components, researchers have proposed several methods to alleviate the noise effect, thereby recognising the signal characteristics. Laakso et al. [14] reconstructed non-uniform measurement signals using polynomial filtering to minimise the effects of noise. He et al. [15] evaluated noise-disturbing problems and proposed a noise-eliminating method for acoustic emission signals. Zhang et al. [16] proposed a modified joint maximum-likelihood estimation algorithm for burst signals. Aliev et al. [17] analysed the effect on estimation errors of the correlation functions of noise signals using traditional correlation analysis algorithms. Sawma et al. [18] proposed an identification method for motor parameters based on LMS, but the fitting accuracy was affected by the noise components. The abovementioned studies are effective for noise reduction in harmonic signals, but they were unable to handle harmonic signals with extensive interference or multiple defects.

**Fig. 1:**
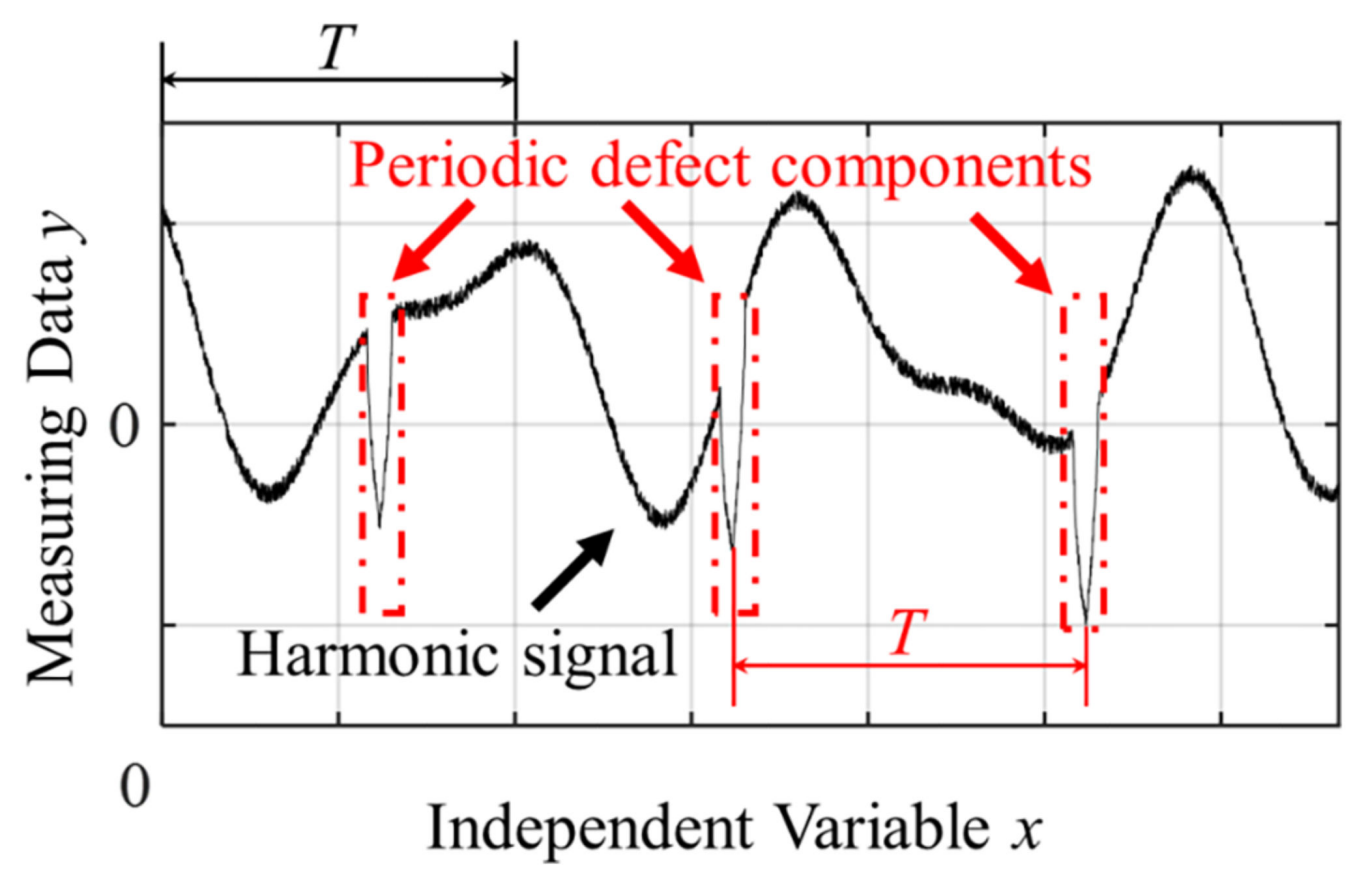
Waveform of harmonic signals that merged with periodic defects.

Owing to the diversity of objects to be measured and the complexity of environmental factors, measurement signals are often merged with one or multiple types of periodic defect components. There are many common real-life examples of harmonic signals merging with obvious periodic defects. For example, 1) two abrupt points appear in the measurement signal of the axle profile owing to the casting joint line; 2) the measurement signal of the rotating cylindrical shaft emerges with multiple pulse signals induced by surface local damage, protuberance, and pit; and 3) the measurement signals of vibration displacement, voltage, and sound decibels are mixed with many transient signals owing to the interference of mechanical, electromagnetic, and even power supply impact excitations. Periodic defects can be easily viewed as valid information of harmonic signals in the measurement process because the span and amplitude of the defect are usually notable. This results in an obvious error in the fitting curve, and the fitting accuracy of the current methods is unsatisfactory. Fig. 1 shows an example of a waveform of harmonic signals merged with periodic defects. As shown in Fig. 1, the amplitude of the defect is large and the defect frequency is periodic.

For measurement signals that emerge with defect components, the most commonly used methods for eliminating defect components are the WT filter [19] and Gaussian process regression (GPR) [20]. However, both methods have shortcomings in terms of effectiveness and accuracy of defect detection. The computational efficiency of the WT filter is costly, and it cannot guarantee quick and accurate calculations simultaneously. GPR is mainly used to deal with the harmonic signals of lose nonperiodic information. When the periodic defect is merged with the measurement signal, GPR cannot distinguish between the defect and non-defect information. Therefore, it is not a suitable method for handling harmonic signals with periodic defects. In addition to the WT filter and GPR methods, researchers have proposed several methods to achieve fault diagnosis. Cheng et al. [21] proposed a noise reduction method based on adaptive weighted symplectic geometry decomposition. However, this method has limitations in defect identification. Mauricio et al. [22] developed a bearing diagnostics method by improving the envelope spectrum, which has high accuracy but more complexity.

The main contribution of this study is the proposal of a novel error-distribution-based selective optimisation method (SOM) to distinguish defects, thereby providing a robust and adaptive approach to fit measurement signals. The proposed SOM can handle large periodic defect components, provide an automatic setting of parameters in the operation variable group, and ultimately eliminate signals that are derived from defect components. Using the proposed SOM, the optimal trigonometric polynomial vector of the harmonic signal can be achieved, which enables an accurate estimation of the equipment’s operational performance and defects.

This study first introduces the SOM model in Section 2. The SOM procedure involves dividing the sample signal into several segments, eliminating partial segments randomly, fitting the residual signal, analysing the coincidence error, and obtaining the optimal trigonometric polynomial vector. Section 2 defines the selection principle for the parameters in the operation variable group for the proposed SOM model. In Section 3, the effectiveness and applicability of the SOM are verified and compared with the current state-of-the-art signal fitting methods, including the OLS, WT, and GPR methods. Section 4 focuses on analysing the association between the characteristic parameters of measurement signals and fitting success probability, and presents the selection principle of operation variable groups. Moreover, the application of the SOM for measuring signals with defect components is verified experimentally in Section 5. Finally, concluding remarks are presented in Section 6.

## 2 Model of selective optimization method

The proposed selective optimisation method (SOM) is a novel signal-processing method based on error distribution that can be used to distinguish defect components from measurement signals. There are three operation variables in the SOM: the number of segments per cycle *N*_seg_, the number of eliminated segments *N*_del_, and the sample size *N*_sec_. The operation variable group (*N*_seg_, *N*_del_, *N*_sec_) was selected according to the signal characteristics, which will be introduced in Section 4.

**Fig. 2:**
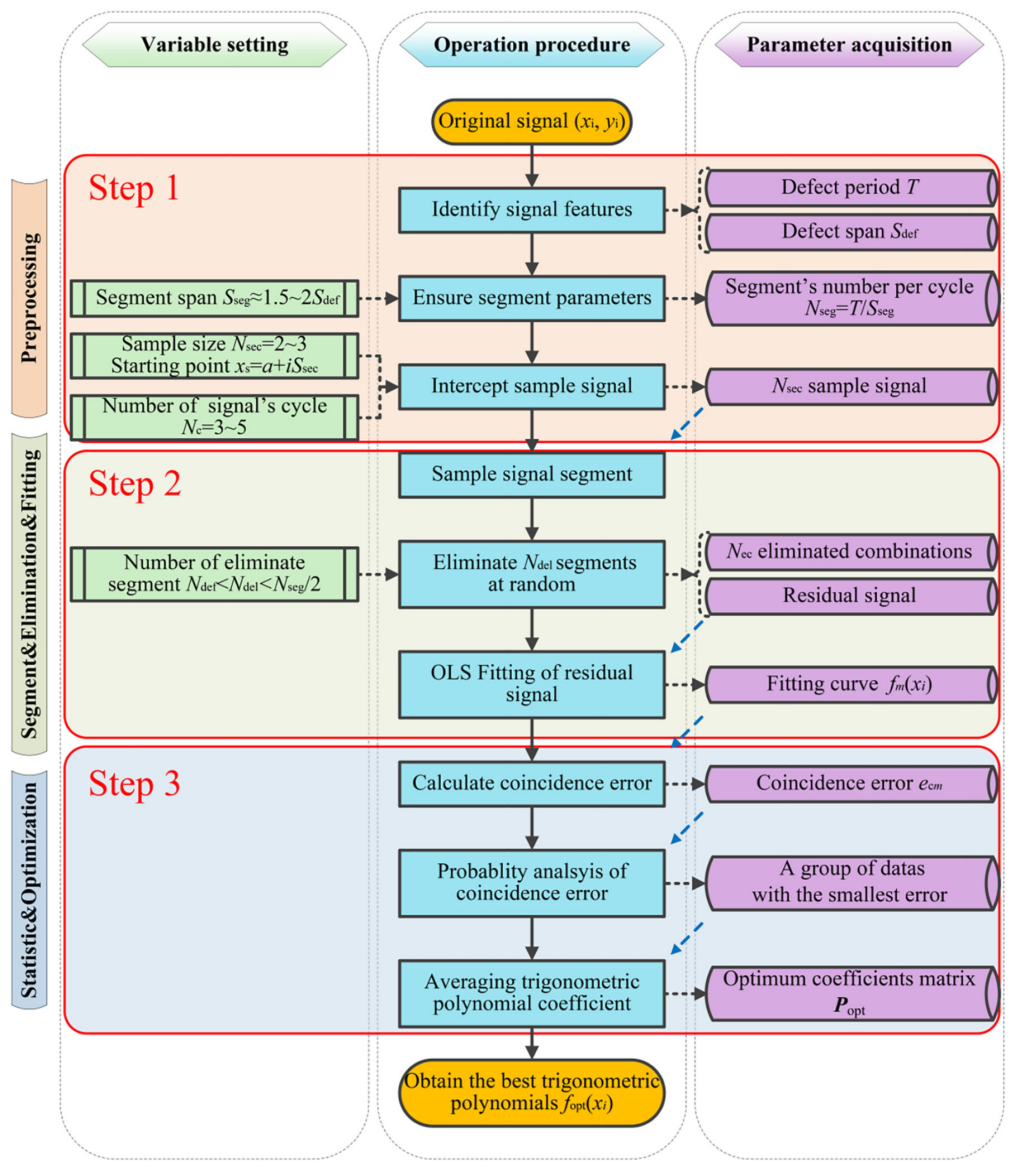
Flow diagram of the selective optimization method.

There are three main steps in the SOM procedure, as shown in the flow diagram in Fig. 2. The first step was signal preprocessing. The operation variables *N*_seg_ and *N*_sec_ are first ensured in this step, and multiple sample signals are intercepted from the measurement signal. The second step is the sample signal segmentation, elimination, and fitting. Operation variables *N*_del_ are ensured, *N*_del_ segments are selected and eliminated randomly, and the residual signal of each eliminated combination is fitted. The last step is statistics and optimisation. The coincidence errors of all the eliminated combinations were calculated and analysed, and the optimum fitting result of the sample signal was obtained. The details of the variable settings, operational procedure, and parameter acquisition are shown in Fig. 2.

### 2.1 Signal preprocessing

The signal preprocessing step involves observing the data and preprocessing the signals into windows for better curve-fitting performance. The defect period *T* and span *S*_def_ were determined based on the measurement signal characteristics. The defect period *T* can be calculated as an integer multiple of the fundamental period of measurement signal, which can be obtained by observing the signal’s peaks and inflexions, or by transforming the measuring variables in the process of signal acquisition. The estimate of the defect span *S*_def_ can be obtained by observing the signal waveform, and the exact value of *S*_def_ can be acquired automatically by analysing the sample signal gradient. The fitting accuracy improves when *S*_def_ < *T*/8. The segment span *S*_seg_ was determined according to *T* and *S*_def_. When the condition of *S*_seg_ > *S*_def_ is satisfied, the number of segments per cycle *N*_seg_ can be calculated as.

(1) $$N_{\text{seg}}=\frac{T}{S_{\text{seg}}}$$

In general, sample size is proportional to the probability of defect elimination. Therefore, several sample signals were intercepted from the measurement signal to aggrandise the sample size *N*_sec_, which is usually set as 2 or 3 to balance the fitting accuracy and efficiency. The acquisition procedure for sample signals can be summarised in the following two steps: 1) a segment is subdivided into *N*_sec_ parts, each part’s span *S*_sec_ = *T*/(*N*_seg_*N*_sec_); 2) *N*_c_ (usually set as 3–5) cycles of data are intercepted starting from each assigned point *x*_s_ of the measurement signal as sample signals, *x*_s_ = *a* + *iS*_sec_, where *a* is the arbitrary moment on the measurement signal, which is usually the initial point of signal acquisition, and *iS*_sec_ is the phase difference of each sample signal’s initial phase, *i* = 0, 1, 2, …, *N*_sec_-1.

**Fig. 3:**
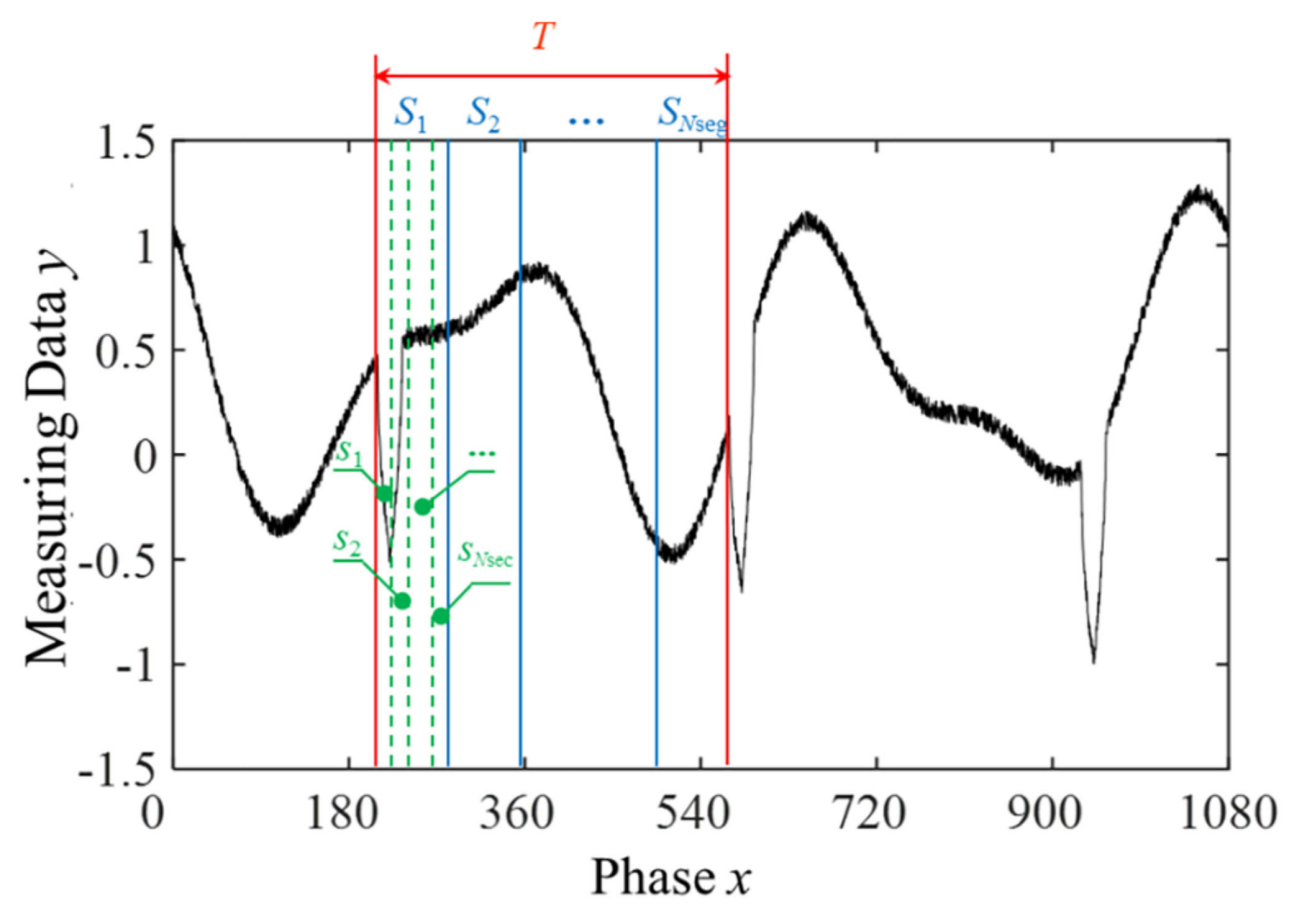
Schematic diagram of signal preprocessing.

The proposed method can handle multiple harmonic signals with various independent variables. To better understand the proposed method, the independent variable *x* was defined as the phase, and *T* was set to 360° in this study. A schematic diagram of the signal preprocessing is shown in Fig. 3. As shown in the figure, *T* is the defect period, and each period of the signal is divided into *N*_seg_ segments, where *S_i_* stands for each segment, *i* = 1,2,…, *N*_seg_. The first segment is divided into *N*_sec_ parts, where *s_i_* stands for each part, *i* = 1,2,…, *N*_sec_. If *S*_seg_ > *S*_def_, the probabilities of a defect contained in one or two adjacent segments are

(2) $$P\left({\text{C}}_{1}\right)=\frac{S_{\text{seg}}-S_{\text{def}}}{S_{\text{seg}}},P\left({\text{C}}_{2}\right)=\frac{S_{\text{def}}}{S_{\text{seg}}}$$

where C_1_ and C_2_ represent the events in which defects occur in one and two adjacent segments, respectively.

### 2.2 Segmentation, elimination, and fitting

The eliminated combination represents the segments of each period, which can be determined according to the first period of sample signals. The principle of elimination was the same for the other periods as it was for the first.

The first period was segmented based on the *N*_seg_ and *S*_seg_ obtained during preprocessing. The *N*_del_ of the *N*_seg_ segments are randomly selected, and *N*_del_ represents the number of eliminated segments. *N*_del_ < *N*_seg_, therefore, the number of eliminated combinations of all sample signals can be written as

(3) $$N_{\text{ec}}=N_{\sec}C_{N_{\text{seg}}}^{N_{\text{del}}},N_{\text{del}}<N_{\text{seg}}$$

Subsequently, the corresponding segments of each eliminated combination were removed. The probabilities of eliminating defects are

(4) $$P\left({\text{D}}_{1}\right)=\frac{N_{\text{del}}}{N_{\text{seg}}},P\left({\text{D}}_{2}\right)=\frac{N_{\text{del}}\left(N_{\text{del}}-1\right)}{N_{\text{seg}}\left(N_{\text{seg}}-1\right)}$$

where D_1_ and D_2_ represent the events in which the defects are eliminated when C_1_ and C_2_ are satisfied, respectively. The probability was significantly reduced when the defect was distributed in two segments. Setting *S*_seg_ > *S*_def_, the probability of the defect being eliminated is

(5) $$P\left(\text{D}\right)=P\left({\text{C}}_{1}\right)P\left({\text{D}}_{1}\right)+P\left({\text{C}}_{2}\right)P\left({\text{D}}_{2}\right)$$

where D represents the case where defects are eliminated. Substituting (1), (2), and (4) into (5), the eliminated probability can be expressed as

(6) $$P\left(\text{D}\right)=\frac{N_{\text{del}}T\left(S_{\text{seg}}-S_{\text{def}}\right)-S_{\text{seg}}N_{\text{del}}\left(S_{\text{seg}}-S_{\text{def}}N_{\text{del}}\right)}{T\left(T-S_{\text{seg}}\right)}$$

There are two operation variables, *N*_seg_ (in the form of *S*_seg_) and *N*_del_ in Eq. (6). *P*(D) increases with *S*_seg_ and *N*_del_, if *T* and *S*_def_ are constant. This result indicates that the probability of defects being eliminated*, P*(D), is positively correlated with the total amount of signal rejection, *N*_del_ × *S*_seg_. However, removing too much data can lead to obvious drawbacks. Therefore, *N*_del_ should be chosen based on the principle *N*_def_ < *N*_del_ < *N*_seg_/2, and *S*_seg_ should be set as *S*_seg_ = *k*_e_*S*_def_ and *k*_e_ ∈ [1.5,2]. If defects are contained in one or two adjacent segments and *S*_seg_ > *S*_def_, the defect can be eliminated completely only if all segments involving defects are eliminated. Based on this combination, there must be several elimination combinations that include segments with defects. The associations between each signal characteristic parameter and the SOM operation variables are analysed in Section 4 (the selection principles of operation variable group are introduced in Section 4.3).

According to the principle of signal elimination in SOM, each period of the sample signal is divided into *N*_seg_ segments, and the *N*_del_ segments are removed according to different elimination combinations. The residual signal is periodic; thus, it can be fitted based on OLS.

OLS is a mathematical optimisation method for estimating the best relationship between the independent and dependent variables. If we assume that (*x_i_*, *y_i_*) is the coordinate of each point’s residual signals and (*x_i_*, *f_m_*(*x_i_*)) is the coordinate of the fitting curve of the residual signal in the *m*th eliminated combination, we have

(7) $$f_{m}\left(x\right)=P_{m}A$$

where ***A*** is a trigonometric polynomial vector, which can be written as ***A*** = [1 cos*k*_1_*x* sin*k*_1_*x* cos*k*_2_*x* sin*k*_2_*x* …]^T^. *k_i_* represents the fluctuation frequency of the signal, *k*_1_ = 2π/*T*, and can be obtained using the frequency acquisition method [23] or by transforming the measurement variables in the signal acquisition process. ***P**_m_* is the undetermined coefficient vector, and ***P**_m_* = [*a*_0*m*_ *a*_1*m*_ *b*_1*m*_ *a*_2*m*_ *b*_2*m*_ …], *m* = 1, 2, …, *N*_ec_.

**Fig. 4:**
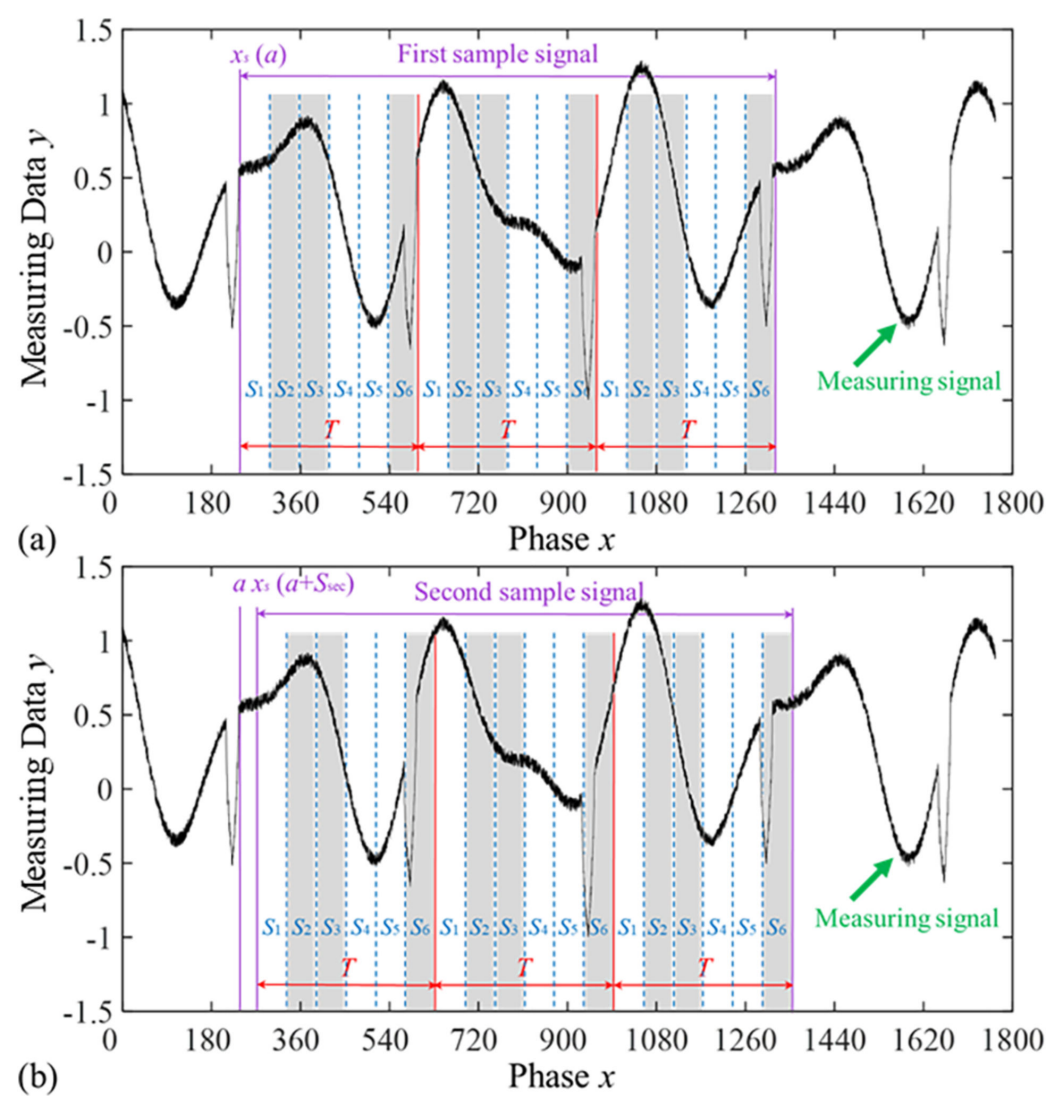
Schematic diagram of the interception, segmentation, and elimination of the sample signal. *N*_seg_ = 6, *N*_del_ = 3, *N*_sec_ = 2, grey bars represent eliminated segments. (a) First sample signal; (b) Second sample signal.

An example is provided in Fig. 4 to demonstrate the interception, segmentation, and elimination of a sample signal using the SOM model. The signal parameters are *T* = 360° and *S*_def_ = 30°, the operation variables are *N*_seg_ = 6, *N*_del_ = 3, and *N*_sec_ = 2, and the grey bars represent the eliminated segments. In this case, the number of elimination combinations is *N*_ec_ = 40, and the probability of eliminating defects in each elimination combination is *P*(D) = 35 %. Various combinations were eliminated to complete the elimination of period defect.

### 2.3 Statistics and optimization of fitting results

**Fig. 5:**
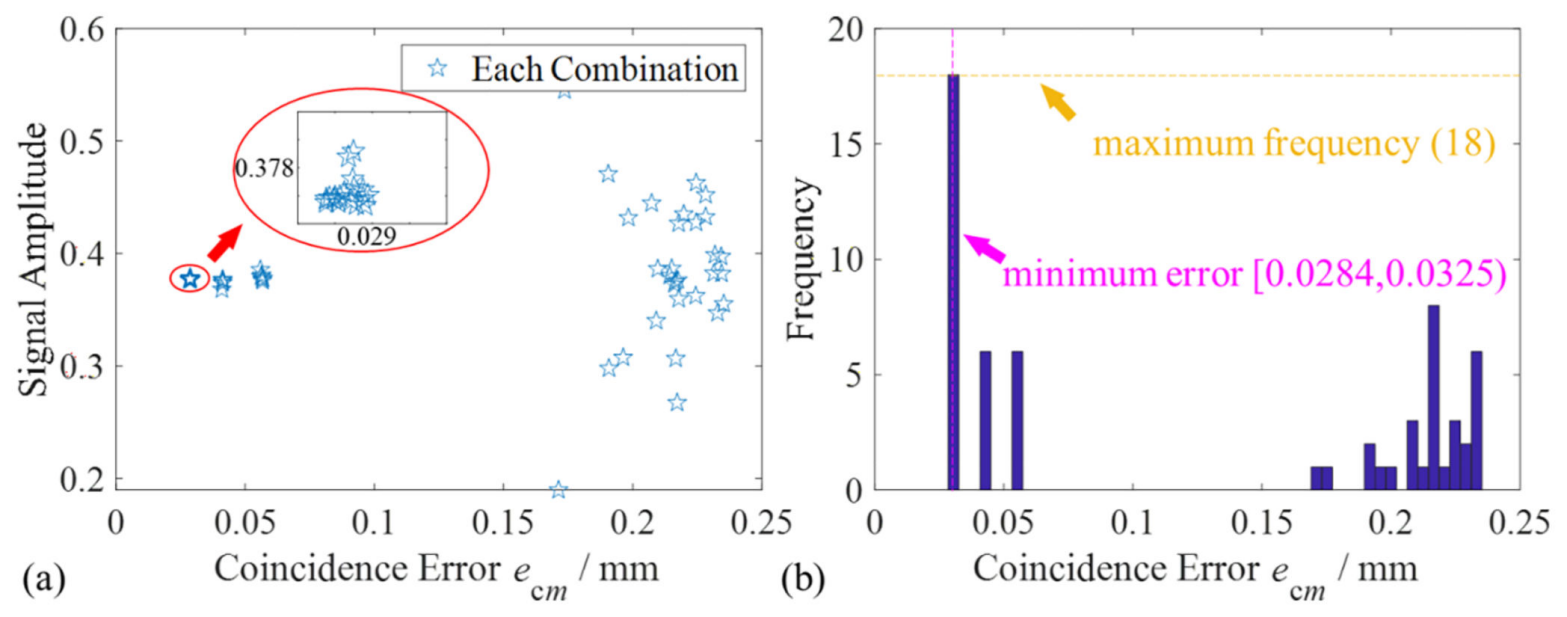
Distribution and statistics of coincidence errors under various eliminated combinations. (a) Distribution and clusters of coincidence error. (b) Frequency histogram of coincidence error.

In the SOM, the coincidence errors between the residual signals and fitting curves are calculated to obtain the optimal fitting trigonometric polynomial of the measurement signal. According to Step 1 and Step 2, *N*_ec_ groups of the fitting curve *f*(*x_i_*) are obtained. The residual sum of the squares of the two curves represents the coincidence error. The coincidence error of the *m*th eliminated combination is defined as

(8) $$e_{cm}=\frac{\sum_{i=1}^{n}{\left(y_{i}-f_{m}\left(x_{i}\right)\right)}^{2}}{N_{\text{c}}},i=1,2,3,\ldots ,n.$$

where *n* represents the number of data points in the residual signal. The *N*_ec_ combination coincidence errors and corresponding signal amplitudes were calculated, and all coincidence errors were evaluated using probability statistics to obtain the optimum trigonometric polynomial. The coincidence error was taken as the abscissa, and the signal amplitude was taken as the ordinate. The distribution of the coincidence errors is shown in Fig. 5 (a), where each star represents the fitting result of each elimination combination. The frequency of some coincidence errors is taken as the ordinate, and the probability statistics of the coincidence errors are shown in Fig. 5 (b). The processed signal and operation variables in Fig. 5 are identical to those in Fig. 4.

**Fig. 6:**
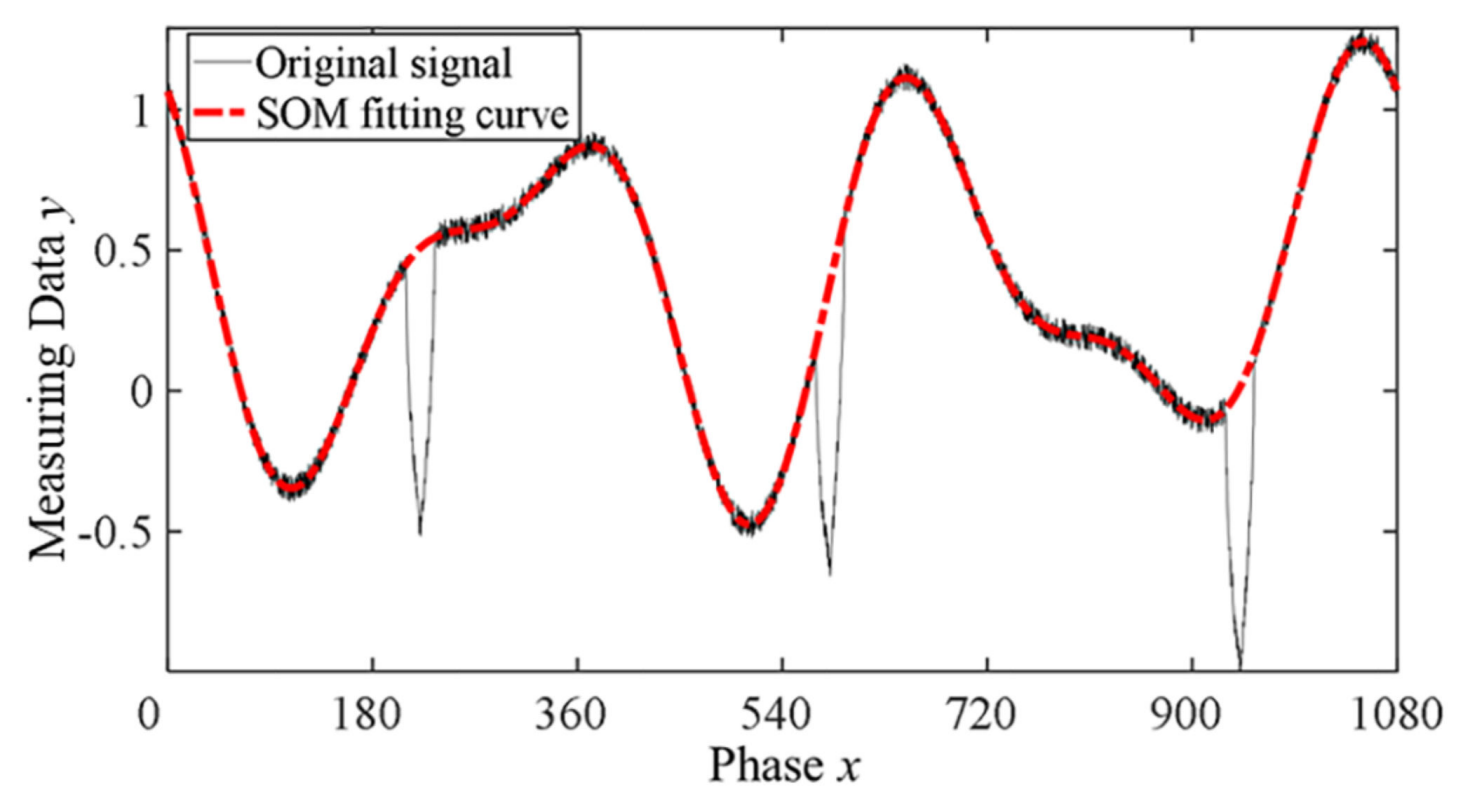
Fitting effect of the selective optimization method.

As shown in Fig. 5 (a), there were three sets of obvious clustering points in the distribution graph, and the coincidence error in these clusters was relatively small. Meanwhile, it can be seen from Fig. 5 (b) that the group of data with the minimum error have the highest frequency. The optimum coefficient vector ***P***_opt_ was obtained by selecting the dataset with the smallest coincidence error and averaging the corresponding trigonometric polynomial coefficients. The SOM fitting function can be expressed as *f*_opt_(*x_i_*) = ***P***_opt_***A***. The fitting results of the harmonic signal in Fig. 4 using SOM are shown in Fig. 6.

## 3 Effectiveness and applicability of SOM

The effectiveness and applicability of the SOM are verified in the following theoretical simulation. The simulation signal can be considered a standard harmonic signal mixed with periodic defects and random noise. The parameters of the standard harmonic signal include the signal amplitude *A*_0_, fluctuation frequency *W*_s_, sampling density of the data acquisition equipment *D*_sd_, and number of sampling signal cycles *N_c_*. Considering SOM’s universality, the signal amplitude *A*_0_ was normalised (i.e. $A_{0}=\sqrt{a_{01}^{2}+b_{01}^{2}}=1$). The defect component parameters include defect amplitude *A*_def_, defect span *S*_def_, and number of defects per cycle *N*_def_. The noise component was ensured according to the noise amplitude, *A*_noise_. The fitting success probabilities of various random signals are calculated to verify the effectiveness of the SOM in this section.

### 3.1 Simulation signal generation

A standard harmonic signal is generated, whose coefficient vector is represented as

(9) $$P_{0}=\left[\begin{array}{llllllll} a_{00} & a_{01} & b_{01} & a_{02} & b_{02} & \ldots & a_{0\text{n}} & b_{0\text{n}} \end{array}\right]$$

**Fig. 7:**
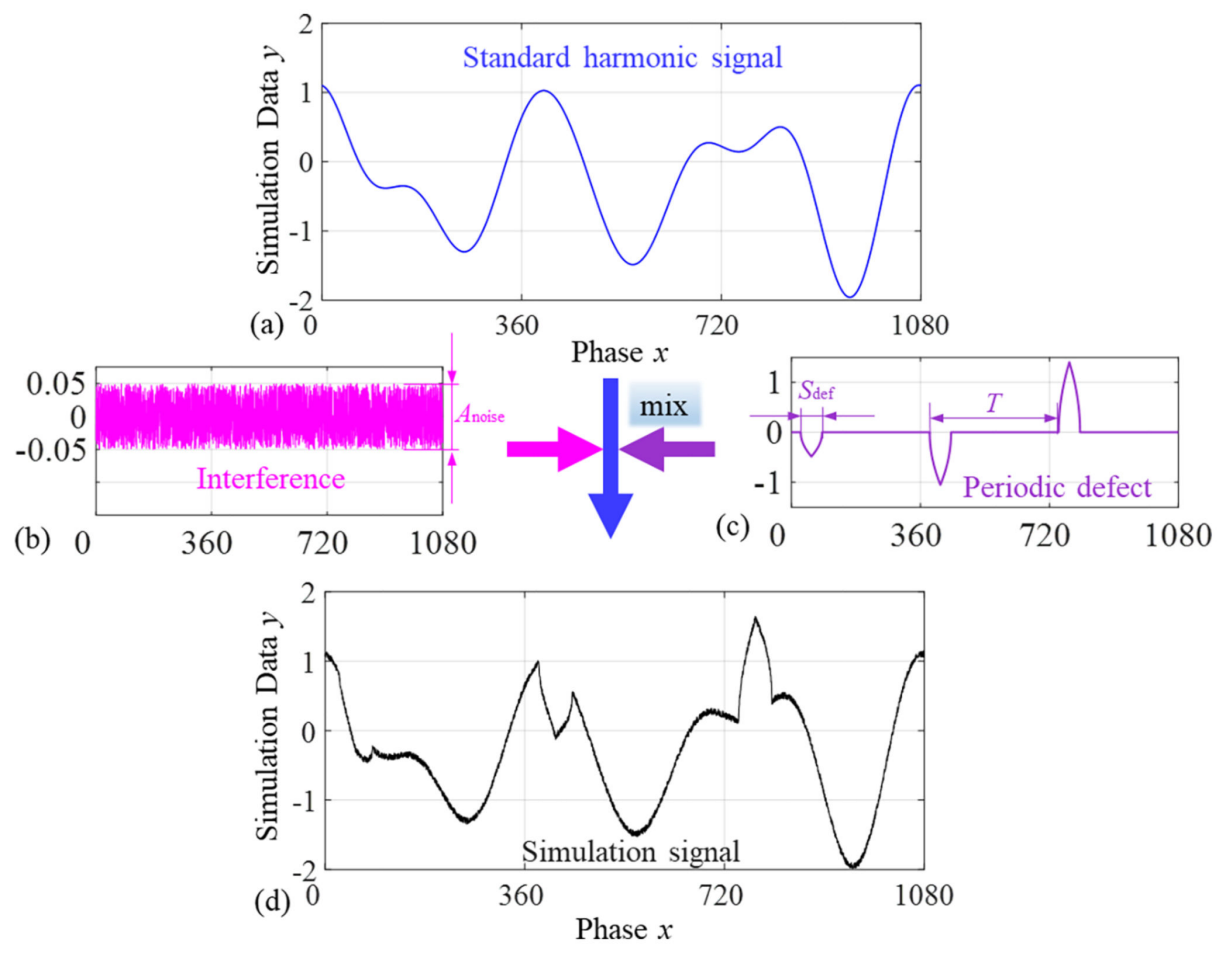
Simulation signal for method verification. (a) Standard harmonic signal; (b) Interference component, *A*_noise_ is noise amplitude; (c) Periodic defect component, *S*_def_ is the defect span; (d) Simulation signal acquired by standard signal mixing with defect and interference components.

Random noise components and periodic defect components were superimposed on the standard harmonic signal. The simulation signal function can be expressed as Eq. (10), and a simulation signal is generated, as shown in Fig. 7.

(10) $$y=a_{00}+a_{01}\cos k_{1}x+b_{01}\sin k_{1}x+a_{02}\cos k_{2}x+b_{02}\sin k_{2}x+\ldots +a_{0\text{n}}\cos k_{\text{n}}x+b_{0\text{n}}\sin k_{\text{n}}x+f_{\text{noise}}\left(x\right)+f_{\text{def}}\left(x\right)$$

where *f*_noise_(*x*) is the added random noise component and *f*_noise_(*x_i_*) ∈ [-*A*_noise_, *A*_noise_]. *f*_def_(*x*) is the added periodic defect component, which is determined by the period *T*, position (initial phase *x*_id_), span *S*_def_, maximum amplitude *A*_def_, and number of cycles *N*_def_. The defect period is equal to the fundamental period of the standard harmonic signal, *N*_def_ is set to 1 or 2, and *x*_id_, *S*_def_, and *A*_def_ are random. Each defect occurs in the simulation signal periodically with different amplitudes, and the function of the defect component is expressed as

(11) $$f_{\text{def}}\left(x\right)=\{\begin{array}{c} R_{n}A_{\text{def}}\sqrt{1-\frac{h\left(x\right)}{h\left(x_{\text{id}}+S_{\text{def}}\right)}}x\in \left[x_{\text{id}}+nT,x_{\text{id}}+S_{\text{def}}+nT\right]\\ 0,x\in \left(x_{\text{id}}+S_{\text{def}}+nT,x_{\text{id}}+\left(n+1\right)T\right) \end{array}$$

where *R_n_* is a random number in the range of [-1,1] and is used to produce periodic defects with different amplitudes; *n* is the *n*th periodic defect, *n* = 1,2,…*N*_c_; and *h*(*x*) is the distance from each point to the defect middle phase, which reflects the shape of the defect hump, $h\left(x\right)=\sqrt{{\left[\cos\left(x\right)-\cos\left(x_{\text{id}}+S_{\text{def}}/2\right)\right]}^{2}+{\left[\sin\left(x\right)-\sin\left(x_{\text{id}}+S_{\text{def}}/2\right)\right]}^{2}}$.

### 3.2 Signal fitting using OLS, SOM, WT and GPR

**Fig. 8:**
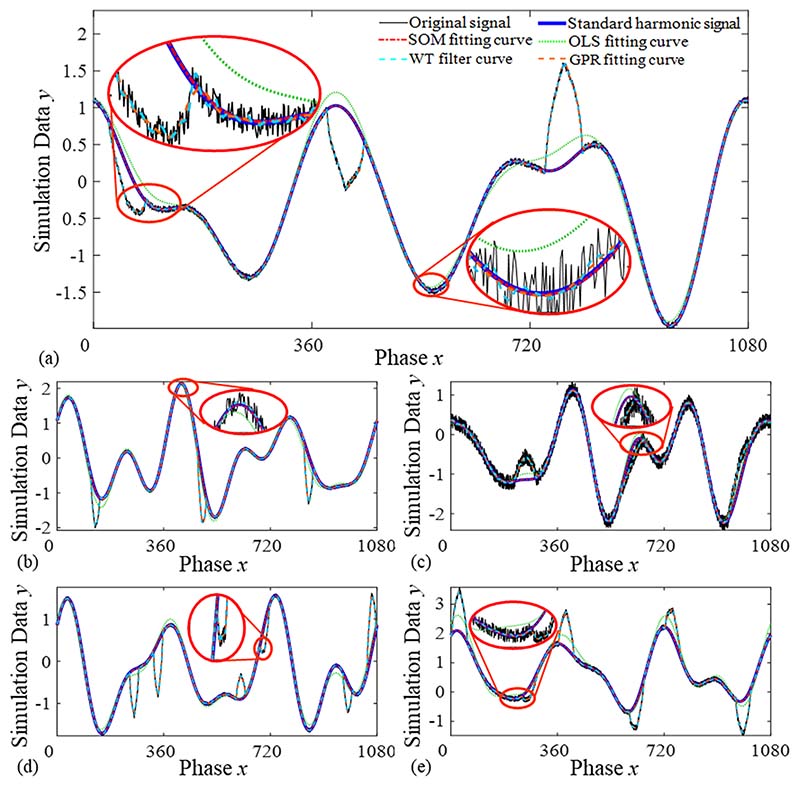
Fitting curves of the harmonic signal with various defect and noise parameters by OLS fitting, SOM fitting, WT filter and GPR fitting. (a) The simulation signal in Fig. 7, *S*_def_ = 60, *N*_def_ = 1, *A*_def_ = 1, *A*_noise_ = 0.05; (b) A small span defect: *S*_def_ = 30, *N*_def_ = 1, *A*_def_ = 1, *A*_noise_ = 0.05; (c) A large span defect with obvious noise: *S*_def_ = 70, *N*_def_ = 1, *A*_def_ = 0.5, *A*_noise_ = 0.2; (d) Two small span defects: *S*_def_ = 30, *N*_def_ = 2, *A*_def_ = 1, *A*_noise_ = 0.05; (e) Two defects with obvious noise: *S*_def_ = 50, *N*_def_ = 2, *A*_def_ = 1, *A*_noise_ = 0.1.

In our experiment, we used four curve-fitting methods: OLS, SOM, WT, and GPR. The fitting parameters of the four methods are defined in this subsection, and their fitting results are compared using an error calculation matrix, as shown in Fig. 8.

For the simulation signals fitted by OLS and SOM, the coefficient vectors of the fitting curve for OLS and SOM can be written as

(12) $$\{\begin{array}{l} P_{\text{OLS}}=\left[\begin{array}{llllllll} a_{\text{e}0} & a_{\text{e}1} & b_{\text{e}1} & a_{\text{e}2} & b_{\text{e}2} & \ldots & a_{\text{en}} & b_{\text{en}} \end{array}\right]\\ P_{\text{SOM}}=\left[\begin{array}{llllllll} a_{\text{p}0} & a_{\text{p}1} & b_{\text{p}1} & a_{\text{p}2} & b_{\text{p}2} & \ldots & a_{\text{pn}} & b_{\text{pn}} \end{array}\right] \end{array}$$

The simulation signals were processed by the WT filter using the MATLAB Wavelet Analyser toolbox. After tuning with different hyperparameters in the WT filter, we chose the Daubechies wavelet with six vanishing moments (the corresponding filter type in MATLAB is ‘db6’) and set the wavelet decomposition to four.

Similar to WT, the simulation signals are fitted by GPR using MATLAB (function ‘fitrgp’). The fit method is set as ‘exact’, the basis function is ‘pureQuadratic’, and the basis matrix is ***H*** = [1, ***X***, ***X***_2_], where $X_{2}=\left[\begin{array}{cccc} x_{11}^{2} & x_{12}^{2} & \ldots & x_{1d}^{2}\\ x_{21}^{2} & x_{22}^{2} & \ldots & x_{2d}^{2}\\ \vdots & \vdots & \vdots & \vdots \\ x_{n1}^{2} & x_{n2}^{2} & \ldots & x_{nd}^{2} \end{array}\right]$.

Fig. 8 (a) shows the curve-fitting results of the simulation signal, which was previously shown in Fig. 7. In Fig. 8 (b)–(e), we provide four typical examples of defect signals and their curve fitting results. These included (b) one small-span defect, (c) a large-span defect with obvious noise, (d) two small-span defects, and (e) two defects with obvious noise.

In Fig. 8, the black lines are simulation signals, the blue curves are standard harmonic signals, and the red, green, cyan, and orange curves represent the fitting results using the SOM, OLS, WT, and GPR methods, respectively. As shown in Fig. 8, the fitting curves of WT and GPR deviate significantly from the standard harmonic signal. This confirms that the WT filter and GPR fitting are not suitable for processing harmonic signals mixed with defect components. Thus, WT and GPR will not be further introduced in subsequent simulations. The fitting curves processed by OLS and SOM are similar to the standard harmonic signal, and the fitting errors and efficiency analyses of the two methods are calculated and compared in the following section.

### 3.3 Efficiency analysis

Numerical simulations were carried out to intuitively compare the fitting success probabilities of OLS and SOM. Without compromising generality, the initial signal characteristic parameters were set as follows: standard harmonic signal parameters, *D*_sd_ = 1024, *W*_s_ = [1, 3/7, 2, 3/4], *N_c_* = 5. The defect component parameters are *S*_def_ ∈ [0,30°], *A*_def_ ∈ [0,5], and *N*_def_ ∈ [0,2]. Noise component parameters: *A*_noise_ [0,0.05].

By calculating the difference between the coefficient vectors of the two fitting methods and that of the simulation signals, the fitting errors of OLS and SOM can be written as

(13) $$\{\begin{array}{c} \delta_{\text{OLS}}=\sqrt{\sum {\left(P_{\text{OLS}}-P_{0}\right)}^{2}}\\ \delta_{\text{SOM}}=\sqrt{\sum {\left(P_{\text{SOM}}-P_{0}\right)}^{2}} \end{array}$$

**Fig. 9:**
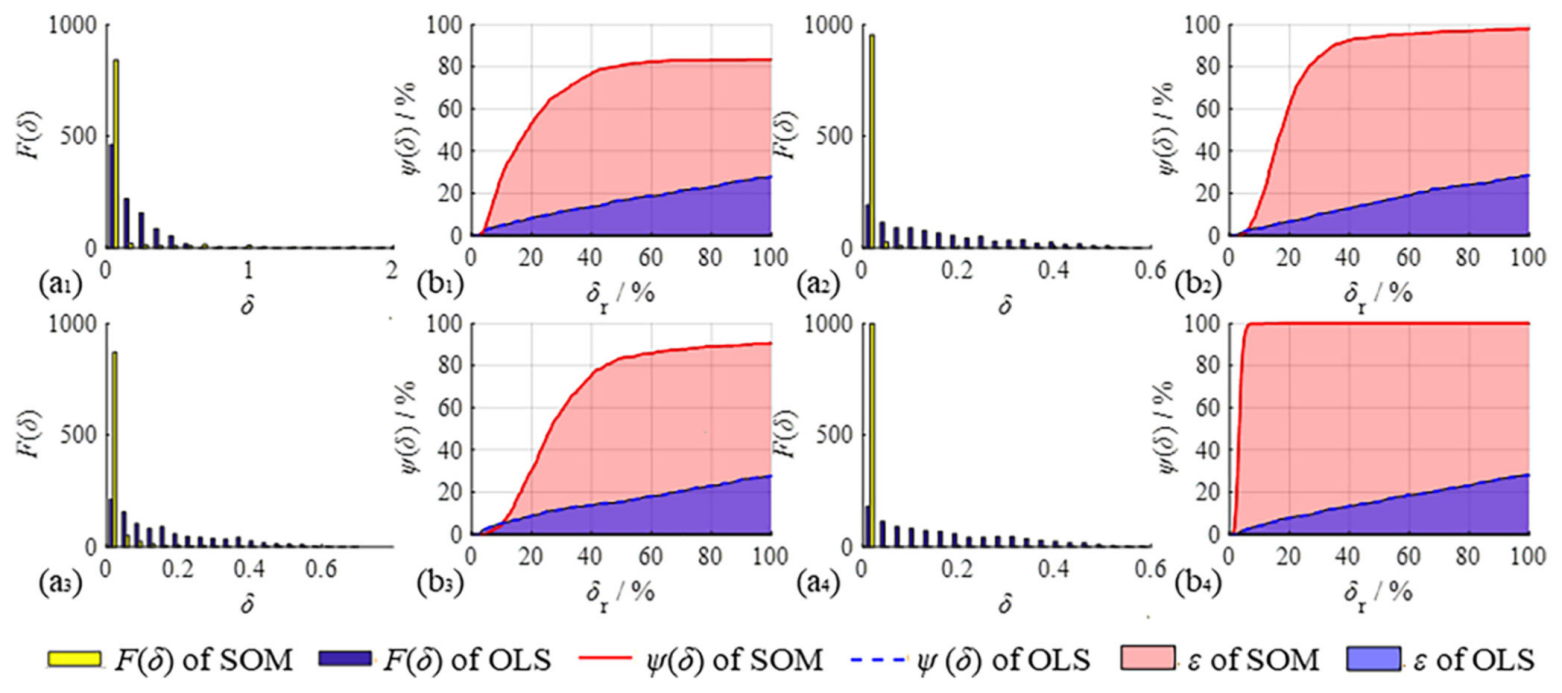
Fitting result statistics in SOM and OLS methods. (a_1-4_) Frequency distribution of fitting error *δ*_r_; (b_1-4_) Fitting success probabilities *ψ*(*δ*_r_). (a_1_) (b_1_): Group 1 (2, 1, 1); (a_2_) (b_2_): group 2 (6, 4, 2); (a_3_) (b_3_): group 3 (8, 6, 3); (a_4_) (b_4_): group 4 (10, 2, 3).

Four groups of operation variables were randomly selected to process the simulation signals to prove the generality of the SOM. The operation variable group (*N*_seg_, *N*_sec_, *N*_del_) was set as group 1: (4, 2, 5), group 2: (6, 4, 2), group 3: (8, 6, 3), and group 4: (10, 2, 3). The influence of the number of simulations *N*_rs_ on the fitting results is not apparent in the process of numerous repeated simulations; therefore, *N*_rs_ is set as 1000 to save simulation time and ensure the reliability of the fitting method. The OLS and SOM fitting errors of each simulation were calculated and analysed, and the frequency distribution of the fitting error *δ* is shown in Fig. 9 (a_1-4_). The abscissa represents the fitting error *δ* and the ordinate represents the corresponding simulation frequency *F*(*δ*) of the fitting error *δ*.

The coordinates are normalised to the range of [0,1], with the total fitting effectiveness of the coordinate system equal to 1 (100 %). As shown in Fig. 9 (b_1-4_), the abscissa is normalised to the relative error *δ*_r_, which is the relative value between the fitting error and the maximum permissible error. Fitting accuracy can be effectively guaranteed when the fitting error is less than *A*_noise_. Thus, the maximum tolerance interval for error was set as *A*_noise_ in this study. The ordinate is normalised to the fitting success probability, representing the relative value of the total frequency below a certain error and the number of repeated simulations *N*_rs_. Therefore, the fitting success probability is expressed as

(14) $$\psi \left(\delta_{\text{r}}\right)=\frac{1}{N_{\text{rs}}}\int_{0}^{\delta_{\text{r}}}F\left(x\right)\text{d}x$$

The fitting success probabilities *ψ*(*δ*_r_) of OLS and SOM are shown in Fig. 9 (b*_i_*). The red and blue lines represent the fitting success probability curves of the two methods, respectively. They can be viewed as typical receiver operating characteristic (ROC) curves. The colour blocks are the area under the curve (AUC), which reflects the method’s total fitting effectiveness *ε* in the range of [0, 1]. The total fitting effectiveness is presented in Eq. (15). To obtain a higher fitting success probability within a small fitting error, the AUC should be as large as possible.

(15) $$\varepsilon \text{=}\frac{1}{A_{\text{noise}}}\int_{0}^{1}\psi \left(\delta_{\text{r}}\right)\text{d}\delta_{\text{r}}$$

The numerical values of each group’s fitting results are shown in Table 1, which includes fitting success probabilities *ψ*(*δ*_r_) when *δ*_r_ = 1 (100 %) and the total fitting effectiveness *ε*.

As shown in Fig. 9 and Table 1, for all four operation variable groups, the fitting results of the simulation signals obtained by SOM were significantly higher than the fitting results of OLS. Both the fitting success probabilities *ψ*(1) and the total fitting effectiveness *ε* of SOM are significantly higher than those of OLS. *ψ*(1) is guaranteed to be greater than 80 % in all groups. This suggests that, when compared to the OLS method, SOM can significantly reduce the influence of defect components and improve fitting accuracy.

In addition, the range of total fitting effectiveness of various operation variable groups is distinctly large, from 65.06 to 96.70, when *ε* of OLS is approximately the same. This indicates that the operation variable groups play an essential role in the total fitting effectiveness, and it is paramount to use the correct group of operation variables to achieve optimal curve fitting results. The association of each signal characteristic parameter with the total fitting effectiveness and the selection principle of operation variable groups is evaluated in Section 4.

## 4 Operation variable selection and optimisation

Because the parameters of the standard harmonic signal and interference are arbitrary, the fitting results of various signals are different in the same operation variable group (*N*_seg_, *N*_del_, *N*_sec_). Therefore, the relationship between the signal characteristics and the optimal operation variable is investigated to acquire the selection principle of (*N*_seg_, *N*_del_, *N*_sec_). In this section, the total fitting effectiveness is represented by the total failure probability *ε*_f_ = 1-*ε*, which shows the fitting effect more intuitively.

### 4.1 Selection of operation variable N_sec_

**Fig. 10:**
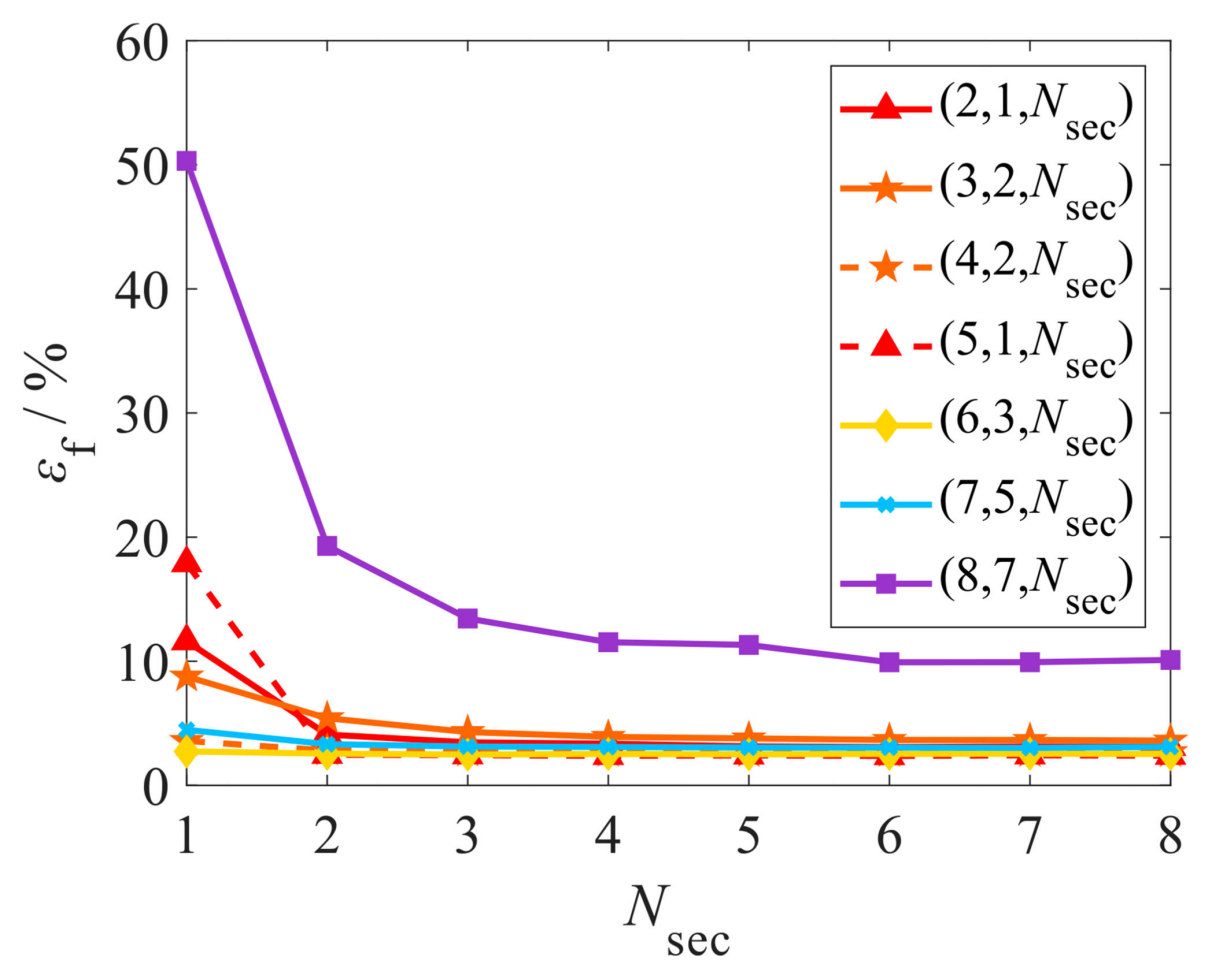
Association of sample size with total failure probability. (*N*_rs_ = 2000, Signal parameters: *D*_sd_ = 1024, *N*_c_ = 5, *N*_def_ = 1, *A*_def_ = 2, *S*_def_ = 30°, *A*_noise_ = 0.01, *W*_s_ = [1, 5/3]).

To analyse the association between sample size *N*_sec_ and fitting effectiveness, the total failure probability *ε*_f_ for different values of *N*_seg_ and *N*_del_ are calculated and shown in Fig. 10. There are seven groups of operation variables: (2, 1, *N*_sec_), (3, 2, *N*_sec_), (4, 2, *N*_sec_), (5, 1, *N*_sec_), (6, 3, *N*_sec_), (7, 5, *N*_sec_), and (8, 7, *N*_sec_). The abscissa represents the sample size (*N*_sec_) and the ordinate is the total failure probability *ε*_f_.

As shown in Fig. 10, the total failure probability *ε*_f_ is significantly high in the case of *N*_sec_ = 1, and the influence of the increase in *N*_sec_ is slight when *N*_sec_ greater than 3. Thus, the situation of *N*_sec_ = 1 should be avoided when setting a generic algorithm, and the selection principle of *N*_sec_ is that *N*_sec_ is usually set to 2 or 3 to ensure higher processing efficiency and fitting accuracy simultaneously.

### 4.2 Selection of operation variables N_seg_ and N_del_

#### 4.2.1 Influence of signal parameters on total failure probability

To explore the influence of each signal parameter on the total failure probability, *N*_sec_ was set to 3 according to the selection principle described in Section 4.1, and the operation variable groups (2, 1, 3) (5, 1, 3) (8, 1, 3) (8, 2, 3) (8, 4, 3) (8, 6, 3) were selected as research objects, considering the influence of *N*_seg_, *N*_del_, and *N*_del_/*N*_seg_ on the total failure probability *ε*_f_.

##### (1) Effect of standard harmonic signal parameters

**Fig. 11:**
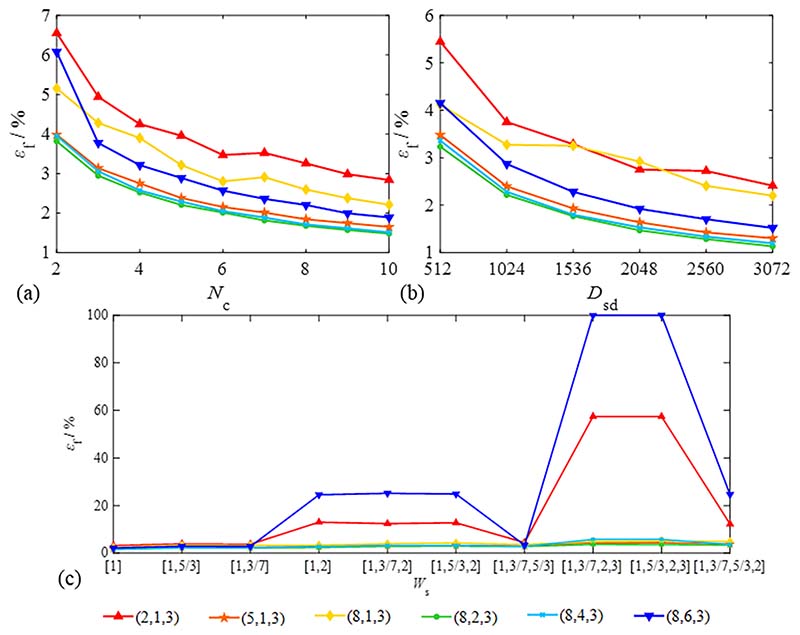
Evaluation of total failure probability based on different standard harmonic signal parameters. (The same parameters: *N*_rs_ = 2000, *S*_def_ = 30°, *N*_def_ = 1, *A*_def_ = 2) (a) The number of sampling cycles *N*_c_ (*D*_sd_ = 1024, *A*_noise_ = 0.005, *W*_s_ = [1,5/3]); (b) sampling density *D*_sd_ (*N*_c_ = 5, *A*_noise_ = 0.005, *W*_s_ = [1,5/3]); (c) Fluctuation frequency *W*_s_ (*D*_sd_ = 1024, *N*_c_ = 5, *A*_noise_ = 0.01).

Standard harmonic signal parameters mainly include the number of sampling cycles *N*_c_, sampling density *D*_sd_, and signal fluctuation frequency *W*_s_, where *N*_c_ and *D*_sd_ determine the data volume, and *W*_s_ determines the harmonic signal waveform. Their influence on the total failure probability *ε*_f_ was calculated, as shown in Fig. 11.

As shown in Fig. 11 (a) and (b), the total failure probability *ε*_f_ will be slightly reduced with increasing data volume for each operation variable group. Compared with *W*_s_, the influences of *N*_c_ and *D*_sd_ are not apparent, and *ε*_f_ can be controlled to less than 5 % in most cases. The *ε*_f_ of each operation variable was similar when *N*_c_ and *D*_sd_ were constant, and the largest difference was 2 %.

The total failure probability *ε*_f_ of (2, 1, 3) and (8, 6, 3) in Fig. 11 (c) is evident for the simulation signal, including the frequency multiplier. This shows that the extraction of valid information is difficult when too many signals are eliminated. Therefore, signal rejection should be minimised as much as possible on the premise that *S*_seg_ > *S*_def_ if harmonic signals are mixed with the frequency multiplier components.

##### (2) Effect of defect component’s parameters

**Fig. 12:**
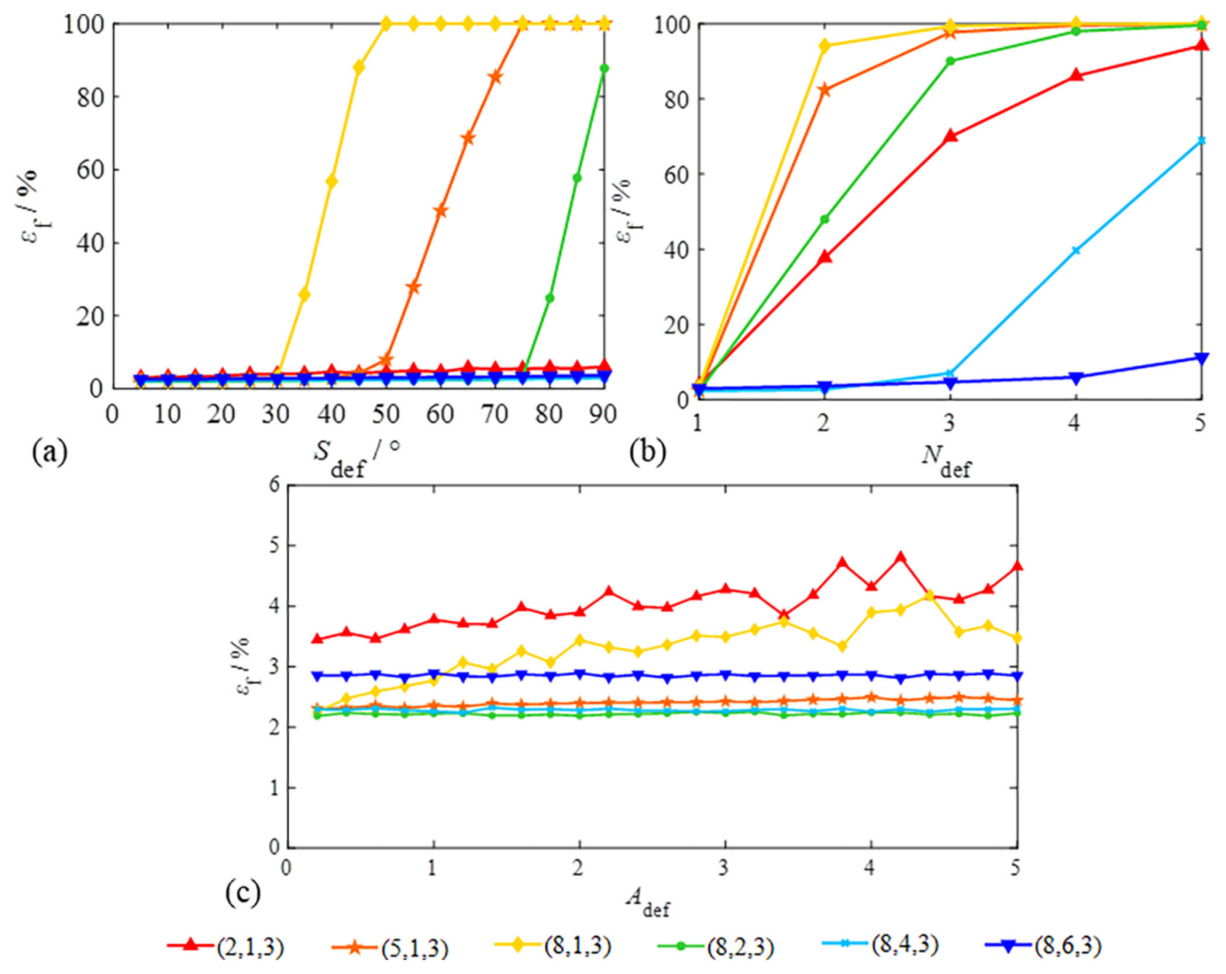
Evaluation of total failure probability based on different defect parameters. (The same parameters: *N*_rs_ = 2000, *D*_sd_ = 1024, *N*_c_ = 5, *A*_noise_ = 0.005, *W*_s_ = [1, 5/3]) (a) defect span *S*_def_ (*N*_def_ = 1, *A*_def_ = 2); (b) defect amplitude *A*_def_ (*N*_def_ = 1, *S*_def_ = 30°); (c) number of defects per cycle *N*_def_ (*A*_def_ = 2, *S*_def_ = 30°).

As the determining factors of the signal waveform, defect parameters (e.g., *S*_def_, *A*_def_ and *N*_def_) should be carefully considered to optimise the selection principle of operation variables. The simulation signal can no longer be regarded as a low-frequency harmonic signal when *S*_def_ > *T*/4; therefore, the research range of *S*_def_ is (0, 90]. Their influence on the total failure probability *ε*_f_ was calculated, as shown in Fig. 12.

As shown in Fig. 12 (a) and (b), the influence of large defects and the number of defects are evident and cannot be ignored. The defect be eliminated efficaciously, only if *S*_seg_ greater than 1.5*S*_def_ and *N*_del_ ≥ *N*_def_ and *ε*_f_ is less than 5 %. In addition, all cases have a high fitting accuracy when *N*_def_ = 1; however, if *N*_def_ ≥ 2, *N*_del_ should be larger than *N*_def_ to obtain a higher fitting accuracy. As shown in Fig. 12 (c), *ε*_f_ increases with *A*_def_ expansion; *ε*_f_ is generally less than 5 %, but *ε*_f_ of the cases *N*_del_ = 1 is larger than in other cases. Fig. 12 shows that the operation variables should satisfy *N*_def_ + 1 ≤ *N*_del_ < *N*_seg_/2.

##### (3) Effect of noise amplitude

**Fig. 13:**
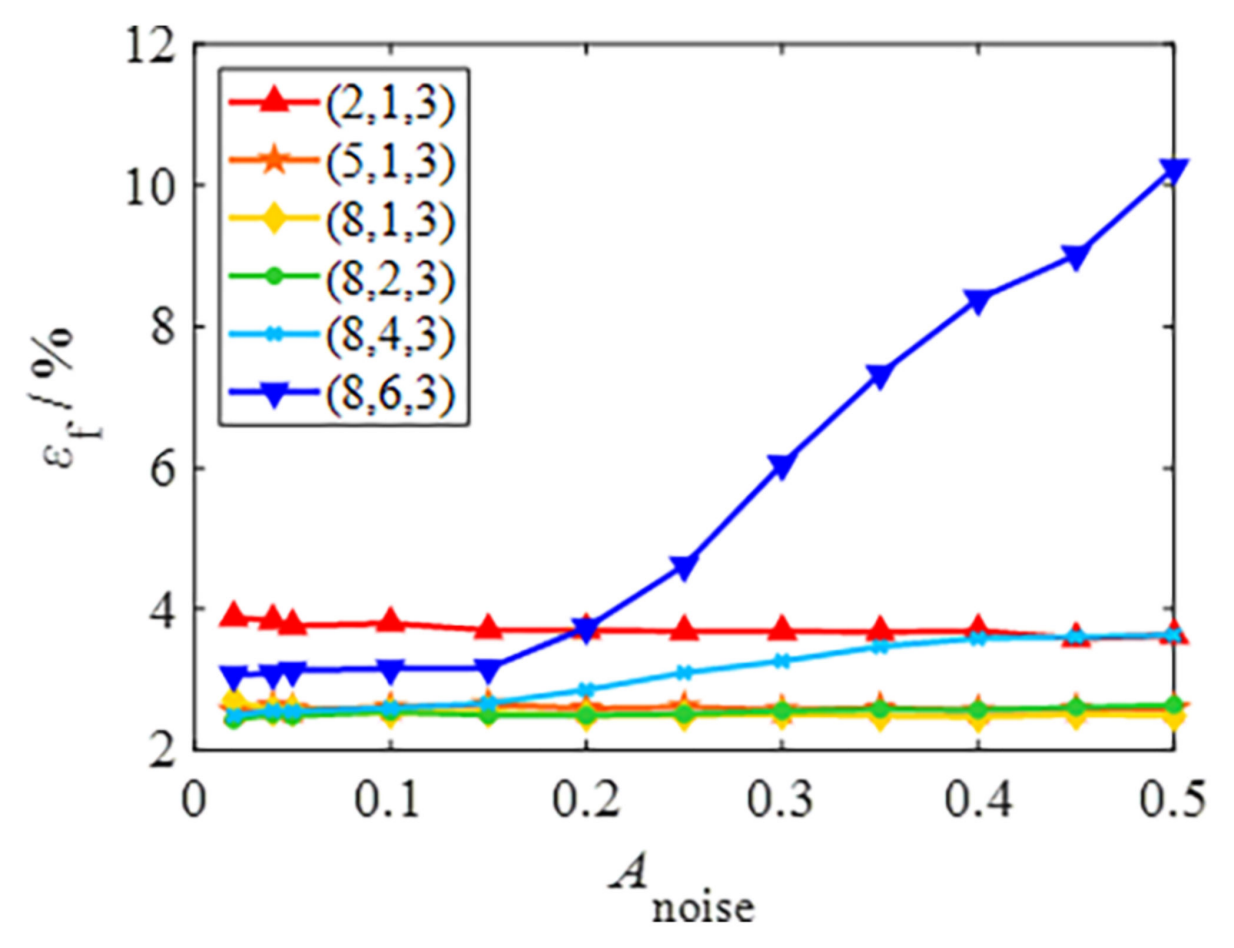
Evaluation of total failure probability based on different noise amplitudes. (Signal parameters: *N*_rs_ = 2000, *D*_sd_ = 1024, *N*_c_ = 5, *W*_s_ = [1,5/ 3], *S*_def_ = 30°, *N*_def_ = 1, *A*_def_ = 2).

Owing to environmental interference, the measurement signals often interfere with various noise components. *A*_noise_ reflects signal stability; the influence of *A*_noise_ on the total failure probability is shown in Fig. 13.

Fig. 13 shows that the changing trend of *ε*_f_ is insignificant except for (8, 6, 3) and (8, 4, 3). This result indicates that the eliminated signal’s span (*N*_del_*S*_seg_) should be less than half of a period (that is, *N*_del_ < *N*_seg_/2) when the noise component is significant.

#### 4.2.2 Orthogonal experiment design of main parameters

According to Section 4.2.1, the influence of *D*_sd_ and *N*_c_ is slight, and the relationship between *N*_def_ and *N*_del_ is given, so they are set as constants, that is, *D*_sd_ = 1024, *N*_c_ = 5, and *N*_del_ = 1. Other parameters *W*_s_, *S*_def_, *A*_def,_ and *A*_noise_ were selected as the main variables to ensure the selection principle of the operation variables *N*_seg_ and *N*_del_. The orthogonal experimental design is shown in Table 2, which includes four factors and three levels.

The operation variables are set as *N*_seg_ = 2,3,…,8, *N*_del_ = 1,2,…,*N*_se*g*_-1, and *N*_sec_ = 3. The calculation results (total failure probability *ε*_f_/%; processing time *t*_p_/ms) for Case 1 are listed in Table 3. All simulation calculations were completed using MATLAB on the same computer (Lenovo, Intel(R) Core(TM) i5-9500 CPU @ 3.00 GHz, RAM 8 GB).

To comprehensively analyse the relationship between the signal parameters and the SOM’s operating variables, the effect of signal parameter variation on processing time *t*_p_ and total failure probability *ε*_f_ were analysed as follows.

##### (1) Computational efficiency analysis

**Fig. 14:**
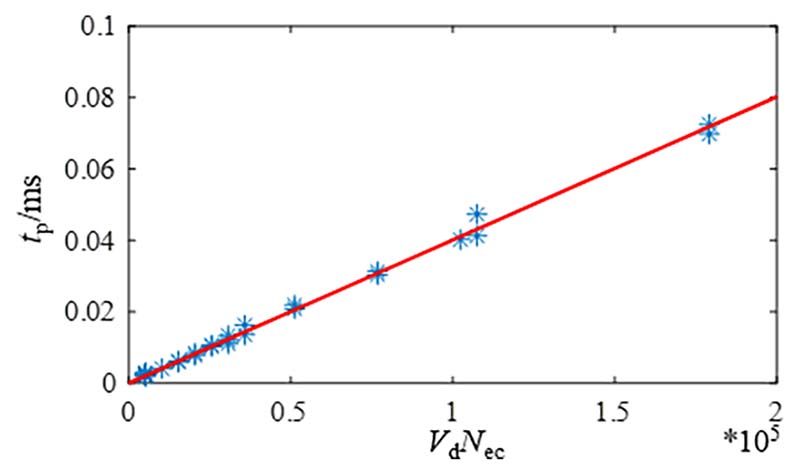
The relationship between processing time and data size of case 1.

In general, the processing time *t*_p_ is related to the number of processed groups and the number of data points per group. Fig. 14 shows that the processing time *t*_p_ of Case 1 was fitted by linear regression.

The processing time *t*_p_ is proportional to the data volume *V*_d_ and the number of eliminated combinations *N*_ec_ by performing a linear regression on the data. The mathematical expression of the linear regression curve is:

(16) $$t_{\text{p}}=c_{\text{f}}V_{\text{d}}N_{\text{ec}}$$

where *V*_d_ is the data volume, *V*_d_ = (1-*N*_del_/*N*_seg_)*N*_c_*D*_sd_, and *c*_f_ is the fitting coefficient of the waveform of simulation signal. The fitting coefficients *c*_f_ of Cases 1 to 9 were calculated, and the results are shown in Table 4.

It can be seen from Table 4 that the processing times of Cases 1–3, Cases 4–6, and Cases 7–9 are marginally the same. Compared with Table 2, the main factor determining the fitting coefficient *c*_f_ is the fluctuation frequency *W*_s_ of the signal. The signal acquisition parameters (*N*_c_ and *D*_sd_) should be selected according to the accuracy requirement for simulation signals with the same fluctuation frequency *W*_s_.

##### (2) Fitting accuracy analysis

**Fig. 15:**
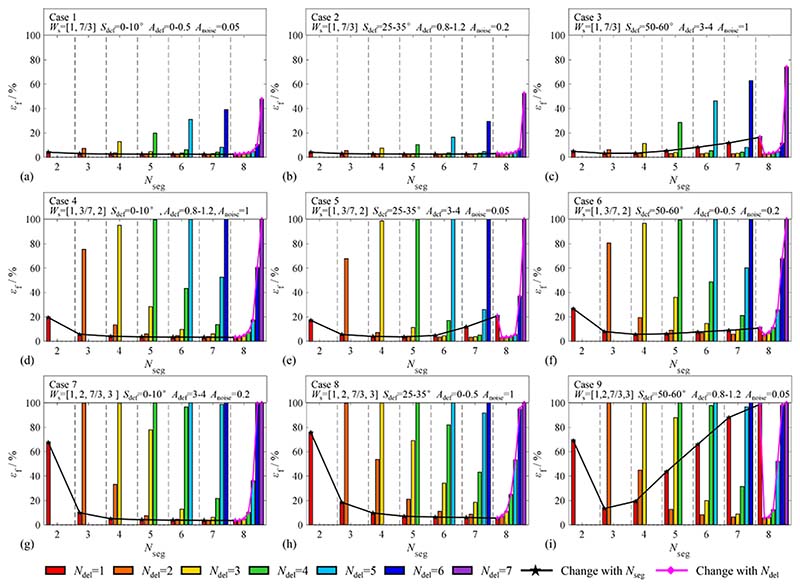
Total failure probability in various operation variable groups. (a)-(i) represent the total failure probability of cases 1–9. The same parameters of each case are *D*_sd_ = 1024, *N*_c=_5, *N*_def_ = 1, the others parameters are: (a) Case 1: *W*_s_ = [1, 7/3], *S*_def_ = 0-10°, *A*_def_ = 0–0.5, *A*_noise_ = 0.05; (b) Case 2: *W*_s_ = [1, 7/3], *S*_def_ = 25-35°, *A*_def_ = 0.8–1.2, *A*_noise_ = 0.2; (c) Case 3: *W*_s_ = [1, 7/3], *S*_def_ = 50-60°, *A*_def_ = 3–4, *A*_noise_ = 1; (d) Case 4: *W*_s_ = [1, 3/7, 2], *S*_def_ = 0-10°, *A*_def_ = 0.8–1.2, *A*_noise_ = 1; (e) Case 5: *W*_s_ = [1, 3/7, 2], *S*_def_ = 25-35°, *A*_def_ = 3–4, *A*_noise_ = 0.05; (f) Case 6: *W*_s_ = [1, 3/7, 2], *S*_def_ = 50-60°, *A*_def_ = 0–0.5, *A*_noise_ = 0.2; (g) Case 7: *W*_s_ = [1, 2, 7/3, 3], *S*_def_ = 0-10°, *A*_def_ = 3–4, *A*_noise_ = 0.2; (h) Case 8: *W*_s_ = [1, 2, 7/3, 3], *S*_def_ = 25-35°, *A*_def_ = 0–0.5, *A*_noise_ = 1; (i) Case 9: *W*_s_ = [1, 2, 7/3, 3], *S*_def_ = 50-60°, *A*_def_ = 0.8–1.2, *A*_noise_ = 0.05;

As mentioned previously, the total failure probability is *ε*_f_ = 1-*ε*. The relationships between each operation variable group (*N*_seg_, *N*_del_, *N*_sec_) and the total failure probability *ε*_f_ are depicted in Fig. 15. Each bar represents an operation variable group (*N*_seg_, *N*_del_, 3). The abscissa represents *N*_seg_ = 2,3,…,8, and the rainbow bars represent *N*_del_ = 1,2,…,7, successively. The ordinate represents the total failure probability, *ε*_f_. The black and pink curves represent the changing trends of *ε*_f_ with increasing *N*_seg_ or *N*_del_, respectively.

As shown in Fig. 15, except for the same signal parameters, *N*_def_ = 1, *D*_sd_ = 1024, and *N*_c_ = 5, there is only one parameter with the same definition value in each row, column, and diagonal. The signal waveform *W*_s_ of the three sub-graphs in each row of Fig. 15 are the same; *ε*_f_ of the first row is less than 5 % in most operation variable groups, and *ε*_f_ increases as the signal order increases. *S*_def_ of each column is the same, and *ε*_f_ of the second column is smaller when *N*_del_*S*_seg_≈*S*_def_, which demonstrates that the signal span of the removed part should be approximately equal to *S*_def_. Beyond that, *A*_def_ of the counterdiagonal and *A*_noise_ of the leading diagonal are the same, and they have less association than *S*_seg_ and *W*_s_.

*S*_seg_ and *W*_s_ are the main factors to be considered in the selection process of the operation variable group. By comparing the *ε*_f_ of the first row and first column, the influence of *W*_s_ should be primarily evaluated if the harmonic signal is mixed with the frequency multiplier. When a large defect exists in the signals, *N*_del_ should be increased appropriately. In most cases, the value of *S*_seg_ should satisfy the conditions *S*_seg_ > *S*_def_ and *S*_seg_ = 1.5 ~ 2*S*_def_ are commonly used. *N*_del_ should be set as small as possible, on the premise that *N*_del_ > *N*_def_. When *S*_def_ is uncertain, regardless of the signal parameters, a larger *N*_seg_ and *N*_del_ = 1/2*N*_seg_ is a good choice.

### 4.3 Selection principles of the optimum operation variable group

This subsection summarises the effects of the signal parameters on the total fitting effectiveness. The research results on the association of signal parameters with fitting effectiveness and suggestions for operation variables are listed in Table 5.

To obtain a higher fitting accuracy, the selection principles of the operation variable group (*N*_seg_, *N*_del_, *N*_sec_) are as follows:

(1) *S*_seg_ should be larger than *S*_def_, and usually *S*_seg_ = *k*_s_*S*_def_, *k*_s_∈(1.5,2], *N*_seg_ = *T/S*_seg_.
(2) The selection of *N*_del_ should ensure complete elimination of defects while preserving as much valid information as possible, usually *N*_def_ + 1 ≤ *N*_del_ < *N*_seg_/2.
(3) *N*_sec_ is proportional to the processing time and fitting accuracy, which are usually set to 2 or 3 to balance their influence.
(4) When harmonic signals are mixed with frequency multiplier components, the amount of signal rejection *N*_del_*S*_seg_ should be minimised as much as possible, on the premise of satisfying principles (1)–(3).

Based on the selection principle, periodic defects are effectively eliminated, and the fitting accuracy of the harmonic signals can be distinctly improved. However, similar to the selection of degree *n* in polynomial curve fitting based on the root mean square error for different data, the fitting errors of various operation variable groups are different; therefore, the coincidence error *e*_cm_ is selected as the judging criterion when seeking the most suitable (*N*_seg_, *N*_del_, *N*_sec_). The smaller the coincidence error, the higher the fitting accuracy.

The operation variable must be chosen according to the operator’s requirements and signal conditions.

In some cases, the span of defect is unknown. We propose an estimation method for the optimum operation variable group to simplify the selection of operation variables. The estimation method is based on minimising the coincidence error *e*_cm_. All operation variable groups that satisfied the selection principles were selected, and their coincidence errors were calculated. The operation variable group with the smallest coincidence error can be approximated as the optimal group for the measurement signal.

## 5 Experiment verification

### 5.1 Measurement signal acquisition

**Fig. 16:**
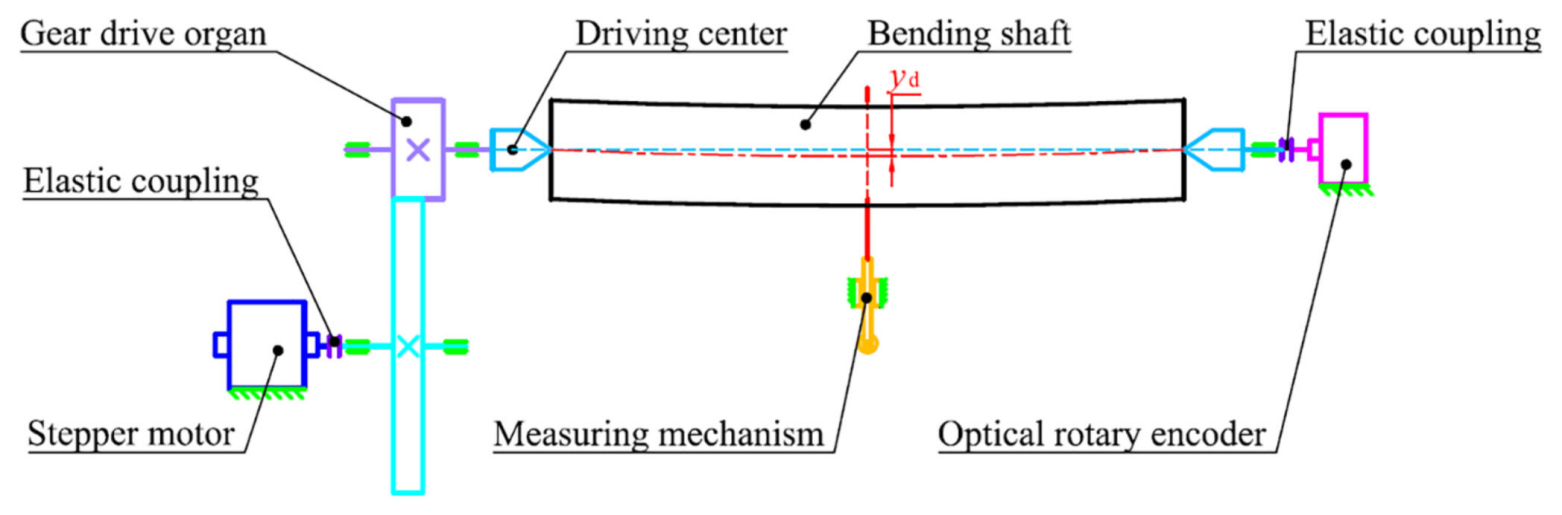
Principle diagram of experimental equipment control and signal acquisition.

In this section, a bending shaft with a small deflection *y*_d_ is used as an example to validate the effectiveness of SOM in real-life engineering applications. When the bending shaft rotates, the measurement signals are regarded as harmonic signals that are resilient to systematic mechanical errors and environmental disturbances. The fundamental period of measurement signal is equal to the rotational period of the bending shaft. The measurement signal of surface damage (e.g. burrs, pits, bumps) can be taken as periodic defect components in harmonic signals, whose period is the same as the rotational period of the bending shaft. As shown in Fig. 16, the bending shaft was clamped on the experimental platform by a pair of centres. The driving force of shaft rotation is transmitted from the stepper motor to the driving centres by a gear drive organ. The fluctuation signal of the bending shaft rotation *y* was measured by the measuring mechanism, and the rotation angle *x* was collected in real time by an optical rotary encoder.

**Fig. 17:**
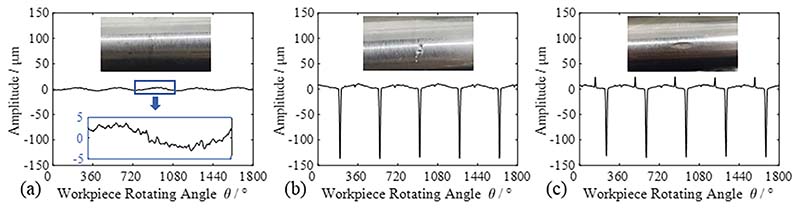
Surface topography and measurement signal of the measured point on the bending shaft. (a) Smooth surface; (b) First damage; (c) Second damage.

To verify the effectiveness of the SOM, we first collected the bending deformation signals of a smooth shaft. The waveform of the measurement signal of the smooth shaft was flat, with only a few noise components. Then, two surface damages were carried out in-house at different positions of the measured outline of the bent shaft in sequence. The deformation signals of the bending shaft for two cases of surface damage were collected. Each cycle of the measurement signal after the first damage included one periodic defect, and the measurement signal after the second damage included two periodic defects. Each measurement experiment was repeated five times. Surface photographs of the measured profiles and the corresponding measurement signals are shown in Fig. 17.

### 5.2 Validation of SOM’s effectiveness

Three groups of measuring signals with zero, one, and two periodic defects were fitted using OLS and SOM, respectively. The trigonometric first-order component *f*_1_(*x*) = *a*_1_cos(*x*) + *b*_1_sin(*x*) of the measurement signals reflects the bending state of the shaft. The first-order amplitude of the measurement signal $A_{1}=\sqrt{a_{1}^{2}+b_{1}^{2}}$ is the deflection of the bending axis of the shaft at the measured point, that is, *A*_1_ = *y*_d_. The fundamental period of the measurement signals is *T*.

**Fig. 18:**
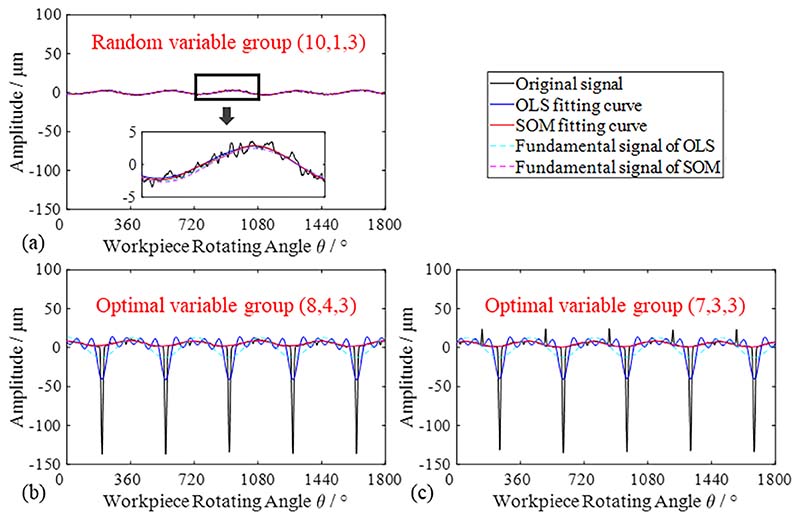
Fitting curves of measurement signal by two methods. (a) Smooth surface; (b) One damage; (c) Two damages.

The fundamental periods of the measurement signals were equal to the rotation period of the bent shaft, where *T* 360°. The measurement signals of the smooth shaft can be approximated as a standard harmonic signal; therefore, the operation variable group can be selected randomly, which is set as (10, 1, 3) in this part, whose fitting curve is shown in Fig. 18 (a). The defect span of the first damage was *S*_def1_≈25°, and the value range of the operation variable group was *N*_seg_∈ [6,9], *N*_del_∈ [1, *N*_seg_/2], and *N*_sec_ = 3. The defect spans of the second damage are *S*_def1_≈25° and *S*_def2_≈15°. The value ranges of the operation variable group are *N*_seg_∈ [6,9], *N*_del_ ∈ [2, *N*_seg_/2], and *N*_sec_ = 3. The coincidence error *e*_cm_ of each operation variable group and corresponding first-order amplitude *A*_1_ are listed in Table 6.

It can be seen from Table 6 that the first-order amplitudes *A*_1_ of the various operation variables are similar. According to the selection principle shown in Section 4.3, the optimal group of measurement signals with one damage and two damages is (8, 4, 3) and (7, 3, 3), respectively. The fitting curves and corresponding first-order curves obtained by the two methods were calculated and are shown in Fig. 18 (b) and (c), respectively.

The fitting results for each measurement signal were calculated and are listed in Table 7. *A*_1_ represents the first-order amplitude of each measurement signal. *A*_mean_ represents the average amplitude of five repeated measurements. *e* is the difference between *A*_1_ of each measurement signal with a defect and *A*_mean_, reflecting the robustness of the fitting method.

As shown in Fig. 18 and Table 7, both OLS and SOM had good fitting effects for the measurement signals of the smooth shaft. However, in the process of fitting measurement signals with defect components, the fitting curves processed by OLS significantly deviate from the normal trajectory, while that of SOM has a high coincidence with measurement signals, which shows that the fitting effect of SOM is more accurate than that of OLS. As shown in Table 7, the fitting error of the measurement signals with defect components using OLS is noteworthy. The fitting effect of the SOM is better than that of OLS, and the fitting error *e*≈1 is approximately equal to the resolution of the sampling devices.

The results in this section show that the fitting accuracy of the SOM is higher than that of OLS. SOM can effectively process measurement signals with periodic defects and precisely obtain the component characteristics of the signal.

## 6 Conclusion

In this study, a novel distribution-based selective optimisation method (SOM) is proposed to effectively process harmonic signals with large periodic defects. SOM is the first and most accurate and effective method based on unsupervised distribution-clustering methods for defect component elimination. The SOM process includes signal segmentation, random elimination of segments, residual signal regression fitting, error distribution statistics, and fitting curve optimisation. The fitting accuracy and computational effectiveness of SOM were verified in a theoretical model and compared with conventional signal fitting methods, including least mean square, wavelet transform-based methods, and Gaussian regression.

The operation variables of the proposed SOM include the segment number, eliminated segment number, and sample size. The associations of the signal characteristic parameters with the fitting effectiveness are investigated, and the selection principle of the operation variables is given in detail. The segment number is related to the defect span. The number of eliminated segments is determined by the segment number and defect number per cycle, and the sample size is usually set to 2 or 3 to balance the fitting accuracy.

Meanwhile, we use a bent shaft with zero, one, and two defects as an example to examine the feasibility of SOM in real-life industrial settings. This experiment confirms that SOM can simultaneously eliminate periodic defect signals from harmonic signals and perform signal regression fitting both accurately and effectively.

In conclusion, this study confirms that the proposed method can effectively eliminate periodic defects and extract standard harmonic components from the measurement signal. It has broad application prospects in mechanical, electronic, instrument, and aerospace industries.

## Figures and Tables

**Table 1:** The fitting results of the SOM and OLS methods.

Group	Operation variables	Fitting success probabilities *ψ*(1)/%		Total fitting effectiveness *ε*/%	
		OLS	SOM	OLS	SOM
1	(2, 1, 1)	27.9	83.3	15.67	67.80
2	(6, 4, 2)	28.4	97.9	15.44	78.37
3	(8, 6, 3)	27.7	90.5	15.60	65.06
4	(10, 2, 3)	28.0	100	15.39	96.70

**Table 2:** Orthogonal experiment design of signal characteristic parameters.

Cases	*D*_sd_	*N*_c_	*N*_def_	*W*_s_	*S*_def_	*A*_def_	*A*_noise_
Case 1	1024	5	1	[1, 7/3]	0-10°	0–0.5	0.05
Case 2	1024	5	1	[1, 7/3]	25°-35°	0.8–1.2	0.2
Case 3	1024	5	1	[1, 7/3]	50°-60°	3–4	1
Case 4	1024	5	1	[1, 3/7, 2]	0-10°	0.8–1.2	1
Case 5	1024	5	1	[1, 3/7, 2]	25°-35°	3–4	0.05
Case 6	1024	5	1	[1, 3/7, 2]	50°-60°	0–0.5	0.2
Case 7	1024	5	1	[1, 2, 7/3,3]	0-10°	3–4	0.2
Case 8	1024	5	1	[1, 2, 7/3,3]	25°-35°	0–0.5	1
Case 9	1024	5	1	[1, 2, 7/3,3]	50°-60°	0.8–1.2	0.05

**Table 3:** Calculation results of case 1 under each operation variable group.

	*N*_del_ = 1	*N*_del_ = 2	*N*_del_ = 3	*N*_del_ = 4	*N*_del_ = 5	*N*_del_ = 6	*N*_del_ = 7
*N*_seg_ = 2	4.15;1.88*	-	-	-	-	-	-
*N*_seg_ = 3	3.03;4.07	7.41;2.16	–	–	–	–	–
*N*_seg_ = 4	2.74;5.89	3.66;6.23	12.82;2.26	–	–	–	–
*N*_seg_ = 5	2.57;7.78	3.04;12.05	4.63;8.41	19.75;2.53	–	–	–
*N*_seg_ = 6	2.60;10.25	2.82;20.83	3.53;21.86	6.18;10.71	31.13;2.31	–	–
*N*_seg_ = 7	2.5;11.06	2.69;30.36	3.21;40.20	4.15;31.29	8.18;13.35	39.15;2.70	–
*N*_seg_ = 8	2.43;13.57	2.65;41.21	3.10;69.80	3.68;72.51	5.09;47.30	10.48;16.22	47.69;3.02

* Each data cell represents the calculation result of each operation variable group (total failure probability *ε*_f_/%; processing time *t*_p_/ms).

**Table 4:** The fitting coefficient value of each case.

	Case 1	Case 2	Case 3	Case 4	Case 5	Case 6	Case 7	Case 8	Case 9
c_f_(*10^-4^)	4.0124	4.0124	4.0056	6.0959	6.1004	6.0977	8.9576	8.7862	8.7429

**Table 5:** Association of signal parameters with the fitting effectiveness.

Signal parameter	Changing trend of fitting success probability with signal parameter increasing	Application suggestions for SOM variables
*N*_c_	Increase slightly	No significant effect
*D*_sd_	Increase slightly	No significant effect
*W*_s_	Decrease	Reduce *N*_del_*S*_seg_
*s*_def_	Decrease	Insure *S*_seg_ > *S*_def_
*A*_def_	Decrease	1 < *N*_del_ < *N*_seg_/2
*N*_def_	Decrease	Insure *N*_del_ > *N*_def_
*A*_noise_	Decrease	Reduce *N*_del_, *N*_del_ < *N*_seg_/2

**Table 6:** Fitting result of each operation variable group.

*N*_seg_	*N*_del_	One damage		Two damage	
		*e*_cm_	*A*_1_	*e*_cm_	*A*_1_
6	1	0.94	3.31	2.51	2.22
6	2	0.71	3.25	0.73	3.12
6	3	0.75	3.70	0.57	3.40
7	1	0.90	3.16	2.46	2.72
7	2	0.57	3.54	0.70	3.23
7	3	0.58	3.74	0.51*	3.48
8	1	0.99	3.39	2.47	2.97
8	2	0.73	3.86	1.03	3.15
8	3	0.54	3.16	0.70	2.73
8	4	0.50*	3.73	0.55	3.13
9	1	1.61	3.70	2.69	3.41
9	2	0.81	2.90	1.89	3.69
9	3	0.52	3.49	0.69	2.51
9	4	0.50	3.61	0.52	3.10

* Minimum coincidence error *e*_cm_ in columns.

**Table 7:** Fitting signal’s amplitude and fitting error of two methods.

Condition	Group	OLS fitting value/*μ*m			SOM fitting value/*μ*m		
		*A*_1_	*A*_mean_	*e*	*A*_1_	*A*_mean_	*e*
Smooth surface	1	2.63	2.57	–	2.51	2.53	–
	2	2.52		–	2.44		–
	3	2.54		–	2.52		–
	4	2.57		–	2.57		–
	5	2.59		–	2.59		–
One damage	1	13.06	13.01	10.49	3.73	3.64	1.20
	2	13.00		10.43	3.30		0.77
	3	12.95		10.38	3.77		1.24
	4	12.94		10.37	3.70		1.17
	5	13.08		10.50	3.69		1.16
Two damage	1	12.74	12.72	10.17	3.48	3.29	1.00
	2	12.84		10.27	3.53		0.95
	3	12.66		10.08	3.24		0.71
	4	12.61		10.04	3.24		0.71
	5	12.74		10.17	2.95		0.42

## Data Availability

The data that has been used is confidential.

## Nomenclature

*ε*: Total effectiveness
*ε*_f_: Total failure probability
*δ*: Fitting error of sample signal
*δ*_OLS_: Fitting error of sample signal by OLS
*δ*_SOM_: Fitting error of sample signal by SOM
*ψ*: Fitting success probability
*a*: Arbitrary moment of the measurement signal
***A***: Trigonometric polynomial vector
*A*_0_: Signal amplitude
*A*_1_: First-order amplitude of measurement signal
*A*_def_: Defect amplitude
*A*_mean_: First-order amplitude’s mean value of five repeat experiments
*A*_noise_: Noise amplitude
*c*_f_: Fitting coefficient
C_1_: The event that defect is involved by one segment
C_2_: The event that defect is involved by two adjacent segments
D: The event that defects are eliminated
D_1_: The event that defects are eliminated when C_1_ is satisfied
D_2_: The event that defects are eliminated when C_2_ is satisfied
*e*_c*m*_: Coincidence error of *m*th eliminated combination
*e*: Error value of each measurement signal’s first-order amplitude
*f_m_*: Fitting data of residual signal in *m*th eliminated combination.
*F*: Frequency of fitting error
*N*_c_: Number of each sampling signal’s cycle
*V*_d_: Data volume
*N*_def_: Number of defects per cycle
*N*_del_: Number of eliminated segments
*N*_ec_: Number of eliminated combinations
*D*_sd_: Sampling density
*N*_rs_: Number of repeated simulations
*N*_sec_: Sample size
*N*_seg_: Number of segments per cycle
*P*(C_1_): Probability of defect contained in one segment
*P*(C_2_): Probability of defect contained in two segments
*P*(D): Probability of defects being eliminated
*P*(D_1_): Probability of a segment being eliminated
*P*(D_2_): Probability of adjacent segments being eliminated simultaneously
***P***_0_: Coefficient vector of the measurement signal
***P***_OLS_: Coefficient vector of OLS fitting curve
***P**_m_*: Coefficient vector of *m*th elimination combination
***P***_opt_: Optimum coefficients vector
***P***_SOM_: Coefficient vector of SOM fitting curve
*S*_def_: Defect span
*S*_sec_: Phase difference of adjacent samples
*S*_seg_: Segment span
*s_i_*: Serial number of each part
*S_i_*: Serial number of each segment
*t*_p_: Processing time
*T*: Defect’s period and harmonic signal’s fundamental period
*W*_s_: Signal fluctuation frequency
*x*_id_: Defect’s initial phase in each period
*x*_s_: Starting points of sample signals
*y*_d_: Deflection of bending shaft
SOM: Selective optimization method
OLS: Ordinary least square
WT: Wavelet transform
GPR: Gaussian process regression
Measurement signal: Signal acquired by sensors or other acquisition devices / the processing object of SOM
Sample signal: Samples intercepted from measurement signal in SOM / the processing result of Step 1 and the object of Step 2
Residual signal: Residual data of measurement signal after signal elimination in SOM / the processing result of Step 2 and the object of Step 3
Simulation signal: Simulation data in the theoretical simulation as measurement signal
Standard harmonic signal: Signal without noise or defect components
